# Taxonomy of *Verrucaria* species characterised by large spores, perithecia leaving pits in the rock and a pale thin thallus in Finland

**DOI:** 10.3897/mycokeys.72.56223

**Published:** 2020-09-02

**Authors:** Juha Pykälä, Annina Kantelinen, Leena Myllys

**Affiliations:** 1 Biodiversity Centre, Finnish Environment Institute, Latokartanonkaari 11, 00790 Helsinki, Finland Finnish Environment Institute Helsinki Finland; 2 Botanical Museum, Finnish Museum of Natural History, P.O. Box 7, FI-00014 University of Helsinki, Finland University of Helsinki Helsinki Finland

**Keywords:** Ascomycota, calcareous rocks, DNA barcoding, Europe, ITS, lichenised fungi, taxonomic revision

## Abstract

Species of *Verrucaria*, characterised by large spores (at least some spores exceeding 25 µm in length), perithecia leaving pits in the rock and a pale thin thallus, form a taxonomically-difficult and poorly-known group. In this study, such species occurring in Finland are revised, based on ITS sequences and morphology. Maximum likelihood analysis of ITS sequence data was used to examine if the species belong to the *Thelidium* group, as suggested by BLAST search. Twelve species are accepted in Finland: *Verrucaria bifurcata***sp. nov.**, *V. cavernarum***sp. nov.**, *V. devergens*, *V. difficilis***sp. nov.**, *V. foveolata*, *V. fuscozonata***sp. nov.**, *V. karelica*, *V. kuusamoensis***sp. nov.**, *V. subdevergens***sp. nov.**, *V. subjunctiva*, *V. subtilis* and *V. vacillans***sp. nov.***Verrucaria foveolata* is nested in *V. subjunctiva* in the phylogeny, but due to morphological and ecogeographical differences, the two taxa are treated as separate species pending further studies. Based on the analysis, the study species belong to the *Thelidium* group. The studied species show a rather high infraspecific morphological, but a low genetic variation. Furthermore, they show considerable overlap in their morphology and many specimens cannot be reliably identified, based on morphology only. All species are restricted to calcareous rocks. *Verrucaria alpigena*, *V. cinereorufa* and *V. hochstetteri* are excluded from the lichen flora of Finland. *Verrucaria grossa* is considered a species with unresolved identity. *Verrucaria foveolata* and *V. subtilis* are rather common on calcareous rocks of Finland while *V. devergens* and *V. kuusamoensis* are restricted to northern Finland. *Verrucaria subjunctiva* occurs mainly in northern Finland. *Verrucaria bifurcata* has been found only from southern Finland. *Verrucaria difficilis* has few localities both in SW and NE Finland. *Verrucaria vacillans* is restricted to calcareous rocks (dolomite) on the mountains of the NW corner of Finland. *Verrucaria fuscozonata*, *V. karelica* and *V. subdevergens* occur only in the Oulanka area in NE Finland. A lectotype is designated for *V. subjunctiva*. The morphology of the Finnish species was compared with 51 European species of *Verrucaria* presumably belonging to the *Thelidium* group.

## Introduction

*Verrucaria* Schrader is a notoriously-difficult group of lichens, which has been proven to be highly polyphyletic (Gueidan et al. 2007, 2009). Numerous species have been previously described from Europe. Due to the high number of described species, one would expect to find a published name for each collected specimen. However, recent studies have shown that this is often not the case. During the past twenty years, twenty-four new species of *Verrucaria* have been described from Europe (Orange 2004, 2013a, 2014; Aptroot and Thüs 2011; Breuss and Berger 2012; Thüs et al. 2015, 2018; Pykälä et al. 2017a, b, 2018, 2019).

Species of *Verrucaria* occurring on calcareous rocks and characterised by pale endolithic or thinly epilithic thallus, large spores (at least some spores exceeding 25 µm in length) and perithecia leaving pits in the rock, form a difficult and poorly-known group of species. Numerous species belonging to this morphogroup have been previously described, mainly from Central and Southern Europe (see, for example, Zschacke 1933; Servít 1948, 1950, 1954). The taxonomy of this morphogroup is highly confusing. Many species have not been reported since their original description and many described species have been supposed to be synonyms or treated as dubious names in need of further study. There is no consensus of the species level taxonomy, but different authors accept different species in this group.

The taxonomy of this morphogroup is rather poorly known also in Fennoscandia and authors have treated the species somewhat differently (see, for example, Vainio 1921; Foucard 2001). The recent Fennoscandian checklist (Nordin et al. 2019) accepts 15 species (potentially) belonging to the group: *V. adelminienii* Zschacke, *V. alpigena* Breuss, *V. caesiopsila* Anzi, *V. cinereorufa* Schaer., *V. devergens* Nyl., *V. dolomitica* (A. Massal.) Kremp., *V. foveolata* (Flörke) A. Massal., *V. grossa* Nyl., *V. hochstetteri* Fr., *V. integra* (Nyl.) Nyl., *V. karelica* Vain., *V. mimicrans* Servít, *V. obscura* Th. Fr. (nom. illeg. non (Sm. & Sowerby) Borrer), *V. papillosa* Ach. and *V. subjunctiva* Nyl. All these species, but *V. obscura*, have been reported from Finland. Four of the species (*V. devergens*, *V. grossa*, *V. karelica* and *V. obscura*) have been described from northern Europe. Thus, only very few species of this morphogroup have been described from northern Europe compared to several dozens of described species from Central Europe. Furthermore, the northern European species have been described a century or more ago and based on somewhat limited field sampling. This suggests that several undescribed species may potentially occur in northern Europe.

The phylogenetic position of large-spored (at least some spores exceeding 25 µm in length) species of *Verrucaria* leaving deep pits in the rock is mainly not known because species of this group are poorly represented in the phylogenetic studies of Verrucariaceae. However, based on the phylogeny of Gueidan et al. (2009), *V. hochstetteri* belongs to the so-called *Thelidium* group. This suggests that species of this morphogroup may belong to the *Thelidium* group.

In this paper, we revise the Finnish species of *Verrucaria* characterised by large spores, thin, predominantly endolithic, thallus and perithecia leaving pits in the rock, using morphology and ITS sequences. We compare the Finnish species with 51 previously-described European species which may presumably belong to the *Thelidium* group, based on their morphology. We also describe seven new species of *Verrucaria* belonging to this group.

## Materials and methods

*Verrucaria* specimens were collected during the large-scale field study of lichens of calcareous rocks and lime quarries in Finland (see Pykälä and Myllys 2016; Pykälä et al. 2017a, b). Type material of 47 relevant species of *Verrucaria* from herbaria B, G, H, H-NYL, M, PRM, S, TUR-V, UPS, VER and W were studied for comparison. Furthermore, the material was compared with four species for which type material could not be located.

### Morphology

Perithecia and thalli were hand-sectioned with razor blades. The sections were examined and measured in tap water. Asci and ascospores were also studied in squash preparations of perithecia mounted in water. Sections and squash preparations of old herbarium specimens were studied using potassium hydroxide (KOH, 10% solution). Additionally, involucrellum characters and exciple colour and diameter were studied by cutting perithecia into two pieces and studying the pieces using a binocular microscope.

The range of ascospore size is indicated as arithmetic mean and standard deviation. Minimum and maximum values are given in parentheses. The size of the perithecia (in diameter) is given in surface view. The colour of the wall of the exciple is the colour of the base of the exciple.

### DNA extraction and sequencing

Total genomic DNA was extracted from perithecia (1–3) of two- to six-year-old herbarium specimens. Most samples were placed in 96-well microplates and sent to the Canadian Centre for DNA Barcoding (**CCDB**). CCDB’s standard protocols (documentation available at http://ccdb.ca/resources.php) were used for extraction, PCR and sequencing. Primers ITS1-LM (Myllys et al. 1999) and ITS4 (White et al. 1990) were used both for PCR and sequencing of the nuclear ribosomal ITS region. The barcode sequences, their trace files along with all relevant collection data and photographs of the voucher specimens were uploaded to the Barcode of Life Data Systems (BOLD, http://www.boldsystems.org) database. The sequences are available in GenBank (see Table 1 for accession numbers).

The DNA of 25 specimens (26865, 29589, 31528, 32606, 33120, 34601, 35326, 35361, 35857, 35920, 35922, 35930, 35933, 35965, 36222, 36244, 36245, 36254, 36294, 36304, 36308, 36335, 36371, 37331, 39475) was extracted using DNeasy Blood & Tissue kit by Qiagen following the protocol described in Myllys et al. (2011). PCR reactions were prepared using PuReTaq Ready-To-Go PCR beads (GE Healthcare). The 25 µl reaction volume contained 19 µl dH2O, 0.4 µM of each primer and 4 µl extracted DNA. PCR was run under the following conditions: initial denaturation for 5 min at 95 °C followed by five cycles of 30 s at 95 °C (denaturation), 30 s at 58 °C (annealing), and 1 min at 72 °C (extension); in the remaining 35 cycles, the annealing temperature was decreased to 56 °C; the PCR schedule ended with a final extension for 7 min at 72 °C. PCR products were cleaned and sequenced by Macrogen Inc., South Korea (www.macrogen.fi). Primers ITS1F (Gardes and Bruns 1993) and ITS4 (White et al. 1990) were used both for PCR amplification and the sequencing of the ITS regions.

### Phylogenetic analyses

The BLAST search facility in GenBank (https://blast.ncbi.nlm.nih.gov/Blast.cgi) was used to find the closest relatives for our material. Based on this search, the studied species are most closely related to *Thelidium umbilicatum* Th. Fr. (95% sequence similarity), *Verrucaria deversa* Vain. (94% sequence similarity) and *Polyblastia abscondita* (Nyl.) Arnold (94% sequence similarity). These species belong to the so-called *Thelidium* group which is morphologically variable with regard to thallus structure, perithecium anatomy, spore pigmentation and spore septation (Gueidan et al. 2007, 2009). Consequently, we included 15 species from this group in our phylogeny (Table 1). *Polyblastia albida* Arnold and *P. fuscoargillacea* Anzi from the *Polyblastia* group were used as outgroup because they are closely related to the *Thelidium* group, based on the phylogeny of Gueidan et al. (2009).

A total of 138 ITS sequences were aligned with MUSCLE v.3.8.31 (Edgar 2004) using EMBL-EBI’s web service (http://www.ebi.ac.uk/Tools/msa/muscle/). The aligned dataset was subjected to Maximum Likelihood analysis (ML). The analysis was performed with RAxML v.8.1.3 (Stamatakis 2014) located at CSC – IT Center for Science (http://www.csc.fi/english). The ITS region was partitioned into ITS1, 5.8S and ITS2. The GTRGAMMA model was used for all partitions. Node support was estimated with 1000 bootstrap replications using the rapid bootstrap algorithm.

**Table 1.** Specimens used in the phylogenetic analyses. New sequences are in bold.

Species	Country	Voucher	GenBank accession numbers
*Polyblastia abscondita*	Sweden	Tibell 23641 (UPS)	EU553507
*P. albida*	Sweden	Savić 3021 (UPS)	EU553492
*P. clandestina*	Sweden	Nordin 5466 (UPS)	EU559740
*P. fuscoargillacea*	Sweden	Palice 7666 (hb. Palice)	EU553498
*P. lutosa*	Sweden	Savić 3163 (UPS)	EU559734
*P. moravica*	Sweden	Savić 3154 (UPS)	EU553522
*P. nidulans*	Sweden	Savić 3015 (UPS)	EU553491
*Staurothele rupifraga*	Sweden	Savić 3003 (UPS)	EU553490
*Thelidium decipiens*	Sweden	Tibell 23959 (UPS)	EU553511
*T. papulare*	UK	Orange 16318 (NMW)	FJ645268
*T. pyrenophorum*	Sweden	Tibell 23649 (UPS)	EU553500
*T. umbilicatum*	Sweden	Tibell 23525 (UPS)	EU559737
*Verrucaria aethiobola*	UK	Orange 16278 (NMW)	FJ664863
*V. aethiobola*	UK	Orange 16309 (NMW)	FJ664864
*V. anziana*	UK	Orange 15898 (NMW)	FJ664829
*V. anziana*	UK	Orange 16103 (NMW)	FJ664830
*V. anziana*	Sweden	Orange 16377 (NMW)	FJ664831
***V. bifurcata***	**Finland**	**Pykälä 33120 (H)**	**MT229719**
***V. bifurcata***	**Finland**	**Pykälä 36722 (H)**	**MT229720**
***V. bifurcata***	**Finland**	**Pykälä 37228 (H)**	**MT229721**
***V. bifurcata***	**Finland**	**Pykälä 45762 (H)**	**MT229722**
*V. calkinsiana*	Canada	McMullin (OAC)	KT695332
***V. cavernarum***	**Finland**	**Pykälä 34527 (H)**	**MT229723**
***V. cavernarum***	**Finland**	**Pykälä 37975 (H)**	**MT229724**
***V. cavernarum***	**Finland**	**Pykälä 41568 (H)**	**MT229725**
*V. deversa*	Sweden	Savić 3063 (UPS)	EU553496
***V. devergens***	**Finland**	**Pykälä 35922 (H)**	**MT229726**
***V. devergens***	**Finland**	**Pykälä 35933 (H)**	**MT229727**
***V. devergens***	**Finland**	**Pykälä 36220 (H)**	**MT229728**
***V. devergens***	**Finland**	**Pykälä 36234 (H)**	**MT229729**
***V. devergens***	**Finland**	**Pykälä 36244 (H)**	**MT229730**
***V. devergens***	**Finland**	**Pykälä 36245 (H)**	**MT229731**
***V. devergens***	**Finland**	**Pykälä 36271 (H)**	**MT229732**
***V. devergens***	**Finland**	**Pykälä 36304 (H)**	**MT229733**
***V. devergens***	**Finland**	**Pykälä 36344 (H)**	**MT229734**
***V. devergens***	**Finland**	**Pykälä 39898 (H)**	**MT229735**
***V. devergens***	**Finland**	**Pykälä 39901 (H)**	**MT229736**
***V. devergens***	**Finland**	**Pykälä 43421 (H)**	**MT229737**
***V. devergens***	**Finland**	**Pykälä 44042 (H)**	**MT229738**
***V. devergens***	**Finland**	**Pykälä 44914 (H)**	**MT229739**
***V. devergens***	**Finland**	**Pykälä 45090 (H)**	**MT229740**
***V. devergens***	**Finland**	**Pykälä 45367 (H)**	**MT229741**
***V. difficilis***	**Finland**	**Pykälä 32687 (H)**	**MT229742**
***V. difficilis***	**Finland**	**Pykälä 39060 (H)**	**MT229743**
***V. difficilis***	**Finland**	**Pykälä 41859 (H)**	**MT229744**
***V. difficilis***	**Finland**	**Pykälä 44811 (H)**	**MT229745**
***V. foveolata***	**Finland**	**Pykälä 31528 (H)**	**MT229746**
***V. foveolata***	**Finland**	**Pykälä 34953 (H)**	**MT229747**
***V. foveolata***	**Finland**	**Pykälä 35395 (H)**	**MT229748**
***V. foveolata***	**Finland**	**Pykälä 35965 (H)**	**MT229749**
***V. foveolata***	**Finland**	**Pykälä 37728 (H)**	**MT229750**
***V. foveolata***	**Finland**	**Pykälä 38119 (H)**	**MT229751**
***V. foveolata***	**Finland**	**Pykälä 38719 (H)**	**MT229752**
***V. foveolata***	**Finland**	**Pykälä 39028 (H)**	**MT229753**
***V. foveolata***	**Finland**	**Pykälä 39294 (H)**	**MT229754**
***V. foveolata***	**Finland**	**Pykälä 40195 (H)**	**MT229755**
***V. foveolata***	**Finland**	**Pykälä 44553 (H)**	**MT229756**
***V. foveolata***	**Finland**	**Pykälä 44952 (H)**	**MT229757**
***V. fuscozonata***	**Finland**	**Pykälä 36222 (H)**	**MT229758**
***V. karelica***	**Finland**	**Pykälä 39625 (H)**	**MT229759**
***V. karelica***	**Finland**	**Pykälä 39991 (H)**	**MT229760**
***V. karelica***	**Finland**	**Pykälä 40235 (H)**	**MT229761**
***V. karelica***	**Finland**	**Pykälä 40325 (H)**	**MT229762**
***V. kuusamoensis***	**Finland**	**Pykälä 35710 (H)**	**MT229763**
***V. kuusamoensis***	**Finland**	**Pykälä 35857 (H)**	**MT229764**
***V. kuusamoensis***	**Finland**	**Pykälä 35920 (H)**	**MT229765**
***V. kuusamoensis***	**Finland**	**Pykälä 36254 (H)**	**MT229766**
***V. kuusamoensis***	**Finland**	**Pykälä 36294 (H)**	**MT229767**
***V. kuusamoensis***	**Finland**	**Pykälä 36335 (H)**	**MT229768**
***V. kuusamoensis***	**Finland**	**Pykälä 39052 (H)**	**MT229769**
***V. kuusamoensis***	**Finland**	**Pykälä 39900 (H)**	**MT229770**
***V. kuusamoensis***	**Finland**	**Pykälä 40219 (H)**	**MT229771**
***V. kuusamoensis***	**Finland**	**Pykälä 44563 (H)**	**MT229772**
***V. kuusamoensis***	**Finland**	**Pykälä 44570 (H)**	**MT229773**
***V. kuusamoensis***	**Finland**	**Pykälä 44694 (H)**	**MT229774**
***V. kuusamoensis***	**Finland**	**Pykälä 44696 (H)**	**MT229775**
***V. kuusamoensis***	**Finland**	**Pykälä 44703 (H)**	**MT229776**
***V. kuusamoensis***	**Finland**	**Pykälä 44744 (H)**	**MT229777**
***V. kuusamoensis***	**Finland**	**Pykälä 44980 (H)**	**MT229778**
***V. kuusamoensis***	**Finland**	**Pykälä 45231 (H)**	**MT229779**
***V. kuusamoensis***	**Finland**	**Pykälä 45330 (H)**	**MT229780**
*V. latebrosa*	Switzerland	Thues W1135	EU249473
*V. latebrosa*	Switzerland	Thues W1097	EU249474
***V. subdevergens***	**Finland**	**Pykälä 39128 (H)**	**MT229781**
***V. subdevergens***	**Finland**	**Pykälä 44550 (H)**	**MT229782**
***V. subdevergens***	**Finland**	**Pykälä 45109 (H)**	**MT229783**
***V. subjunctiva***	**Finland**	**Pykälä 35326 (H)**	**MT229784**
***V. subjunctiva***	**Finland**	**Pykälä 35361 (H)**	**MT229785**
***V. subjunctiva***	**Finland**	**Pykälä 35930 (H)**	**MT229786**
***V. subjunctiva***	**Finland**	**Pykälä 36308 (H)**	**MT229787**
***V. subjunctiva***	**Finland**	**Pykälä 36371 (H)**	**MT229788**
***V. subjunctiva***	**Finland**	**Pykälä 37746 (H)**	**MT229789**
***V. subjunctiva***	**Finland**	**Pykälä 39475 (H)**	**MT229790**
***V. subjunctiva***	**Finland**	**Pykälä 39478 (H)**	**MT229791**
***V. subjunctiva***	**Finland**	**Pykälä 39491 (H)**	**MT229792**
***V. subjunctiva***	**Finland**	**Pykälä 39803 (H)**	**MT229793**
***V. subjunctiva***	**Finland**	**Pykälä 40284 (H)**	**MT229794**
***V. subjunctiva***	**Finland**	**Pykälä 42392 (H)**	**MT229795**
***V. subjunctiva***	**Finland**	**Pykälä 42406 (H)**	**MT229796**
***V. subjunctiva***	**Finland**	**Pykälä 42419 (H)**	**MT229797**
***V. subjunctiva***	**Finland**	**Pykälä 42510 (H)**	**MT229798**
***V. subjunctiva***	**Finland**	**Pykälä 44671 (H)**	**MT229799**
***V. subjunctiva***	**Finland**	**Pykälä 44734 (H)**	**MT229800**
***V. subjunctiva***	**Finland**	**Pykälä 44881 (H)**	**MT229801**
***V. subtilis***	**Finland**	**Pykälä 26865 (H)**	**MT229802**
***V. subtilis***	**Finland**	**Pykälä 29589 (H)**	**MT229803**
***V. subtilis***	**Finland**	**Pykälä 32606 (H)**	**MT229804**
***V. subtilis***	**Finland**	**Pykälä 32749 (H)**	**MT229805**
***V. subtilis***	**Finland**	**Pykälä 34601 (H)**	**MT229806**
***V. subtilis***	**Finland**	**Pykälä 35093 (H)**	**MT229807**
***V. subtilis***	**Finland**	**Pykälä 36819 (H)**	**MT229808**
***V. subtilis***	**Finland**	**Pykälä 37102 (H)**	**MT229809**
***V. subtilis***	**Finland**	**Pykälä 37329 (H)**	**MT229810**
***V. subtilis***	**Finland**	**Pykälä 37331 (H)**	**MT229811**
***V. subtilis***	**Finland**	**Pykälä 37794 (H)**	**MT229812**
***V. subtilis***	**Finland**	**Pykälä 38140 (H)**	**MT229813**
***V. subtilis***	**Finland**	**Pykälä 39870 (H)**	**MT229814**
***V. subtilis***	**Finland**	**Pykälä 40280 (H)**	**MT229815**
***V. subtilis***	**Finland**	**Pykälä 40596 (H)**	**MT229816**
***V. subtilis***	**Finland**	**Pykälä 40833 (H)**	**MT229817**
***V. subtilis***	**Finland**	**Pykälä 40859 (H)**	**MT229818**
***V. subtilis***	**Finland**	**Pykälä 40874 (H)**	**MT229819**
***V. subtilis***	**Finland**	**Pykälä 41857 (H)**	**MT229820**
***V. subtilis***	**Finland**	**Pykälä 42225 (H)**	**MT229821**
***V. subtilis***	**Finland**	**Pykälä 42540 (H)**	**MT229822**
***V. subtilis***	**Finland**	**Pykälä 44843 (H)**	**MT229823**
***V. subtilis***	**Finland**	**Pykälä 44844 (H)**	**MT229824**
***V. subtilis***	**Finland**	**Pykälä 45794 (H)**	**MT229825**
***V. subtilis***	**Finland**	**Pykälä 45817 (H)**	**MT229826**
***V. subtilis***	**Finland**	**Pykälä 45847 (H)**	**MT229827**
***V. vacillans***	**Finland**	**Pykälä 43058 (H)**	**MT229828**
***V. vacillans***	**Finland**	**Pykälä 43118 H)**	**MT229829**
***V. vacillans***	**Finland**	**Pykälä 43232 (H)**	**MT229830**
***V. vacillans***	**Finland**	**Pykälä 43272 (H)**	**MT229831**
***V. vacillans***	**Finland**	**Pykälä 43296 (H)**	**MT229832**
***V. vacillans***	**Finland**	**Pykälä 43302 (H)**	**MT229833**
***V. vacillans***	**Finland**	**Pykälä 43384 (H)**	**MT229834**
***V. vacillans***	**Finland**	**Pykälä 44075 (H)**	**MT229835**
***V. vacillans***	**Finland**	**Pykälä 44081 (H)**	**MT229836**
***V. vacillans***	**Finland**	**Pykälä 44081b (H)**	**MT229837**

## Results

We obtained 119 new nuITS sequences in this study (Table 1). The topology of the ML tree obtained from the ITS dataset is shown in Fig. 1. The Finnish specimens were divided into eleven strongly-supported lineages of which seven are here described as new species: *V. bifurcata*, *V. cavernarum*, *V. difficilis*, *V. fuscozonata* (represented by only one specimen), *V. subdevergens*, *V. kuusamoensis* and *V. vacillans* (see also Fig. 2). In addition to our new species, *V. subtilis*, *V. devergens* and *V. karelica* are monophyletic, whereas the monophyly of either *V. foveolata* or *V. subjunctiva* could not be recovered. Instead, the two species together form a strongly-supported group.

**Figure 1.**
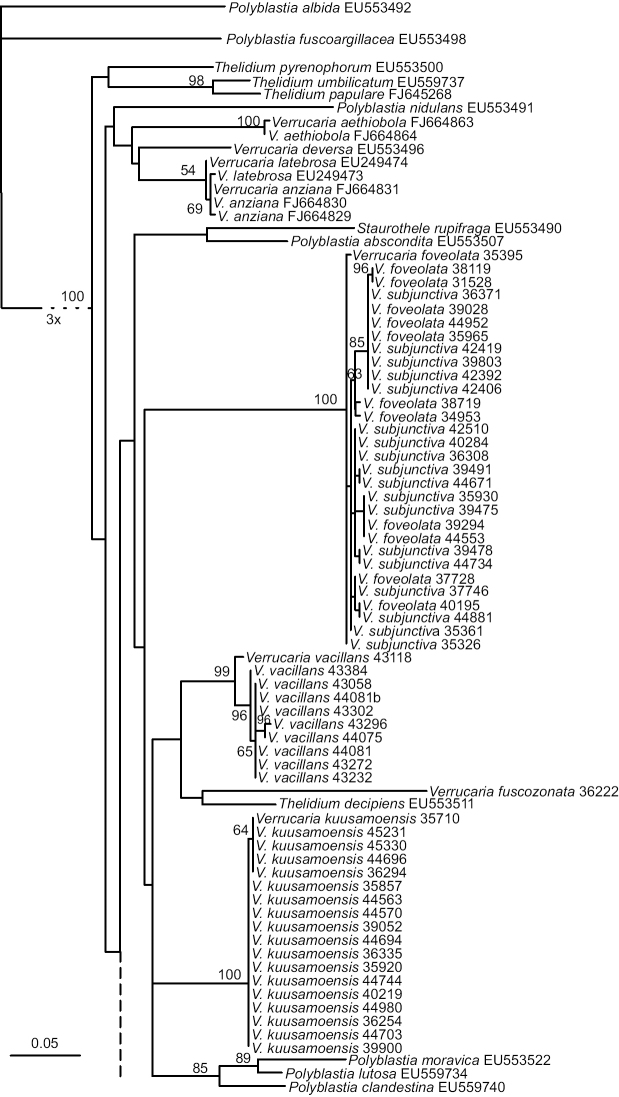
Phylogenetic relationships of *Verrucaria* with large spores, perithecia leaving pits in the rock and pale thin thallus belonging to the *Thelidium* group. A Maximum Likelihood phylogram obtained from the RAxML analysis is based on the ITS dataset. Bootstrap values (> 50%) are shown at nodes. The node leading to the ingroup is shortened and is in reality three times longer.

**Figure 1.**
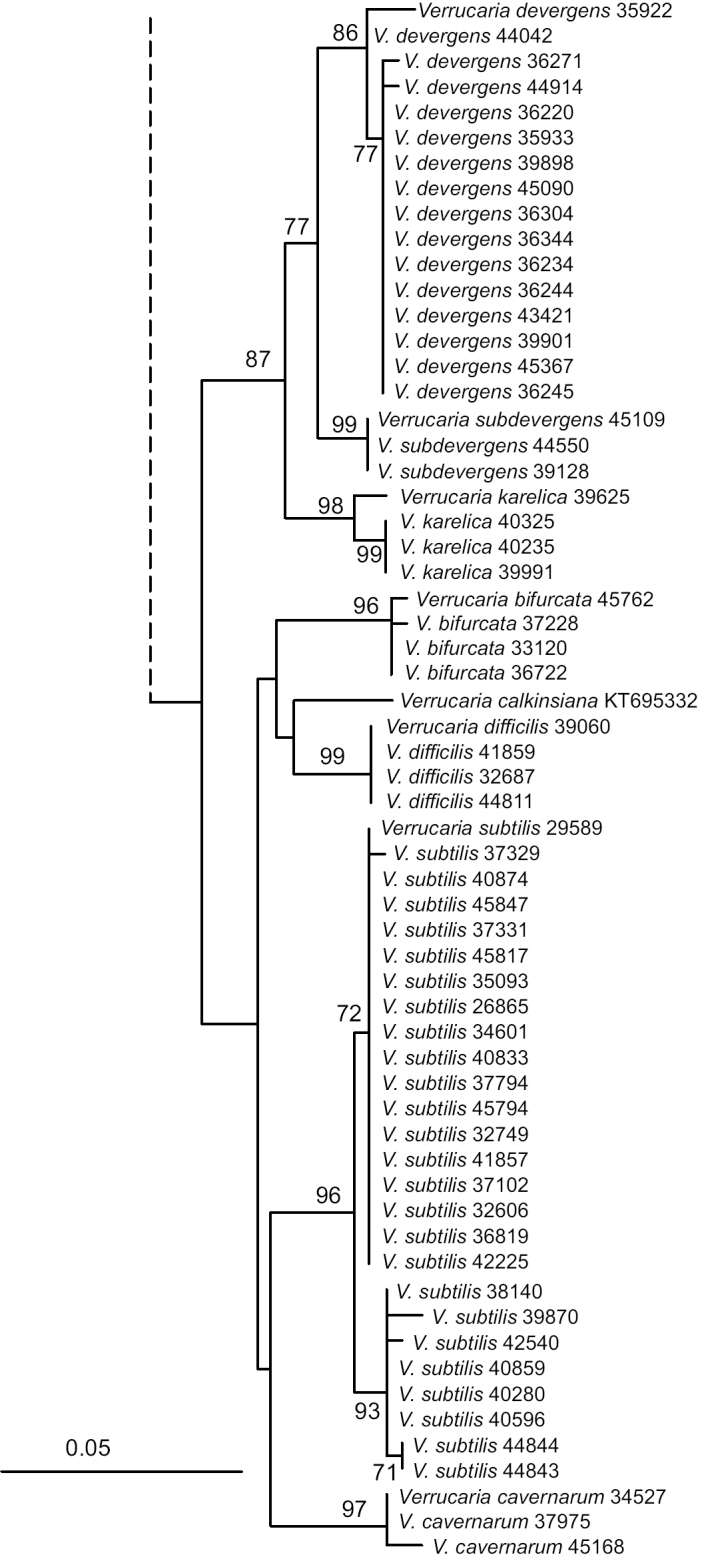
Continued.

**Figure 2.**
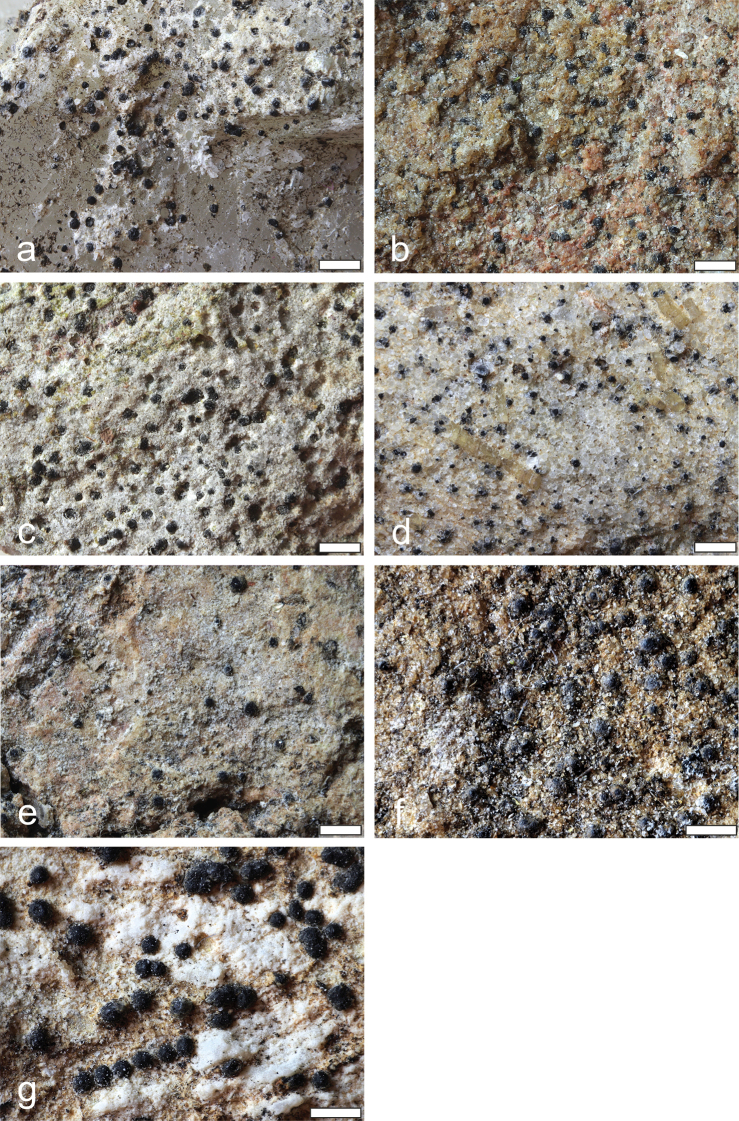
Habitus of the new *Verrucaria* species **A***V. bifurcata* (holotype) **B***V. cavernarum* (holotype) **C***V. difficilis* (holotype) **D***V. fuscozonata* (holotype) **E***V. kuusamoensis* (holotype) **F***V. subdevergens* (holotype) **G***V. vacillans* (holotype). Scale bars: 1 mm (**A–D**), 0.5 mm (**E–G**).

The monophyly of the ingroup was strongly supported, which suggests that all Finnish species in our study are members of the *Thelidium* group *sensu*Gueidan et al. (2009). However, the ITS phylogeny was otherwise poorly resolved. The relationships between the Finnish species remained mostly unclear and only one strongly-supported group was detected: *V. karelica*, *V. subdevergens* and *V. devergens* form a clade. *Verrucaria bifurcata*, *V. difficilis*, *V. cavernarum* and *V. subtilis* also group together, but without any support. *V. calkinsiana* collected in Canada also belongs in this latter clade.

All the studied species had relatively-low infraspecific genetic variation in their ITS sequences, but there seems to be species-specific variation (Table 2). The highest variation was detected in *V. foveolata* with 98.5% sequence similarity. In *V. difficilis* (n = 4) and *V. subdevergens* (n = 3), the sequences were completely identical between the specimens. For comparison, the maximum sequence similarity between closely related *V. devergens* and *V. subdevergens* was 98.7%, but the two species can be separated by the size of the involucrellum (see below for Taxonomy).

**Table 2.** Minimum infraspecific sequence similarity of the ITS region of the species. n = number of studied specimens.

	n	Minimum sequence similarity
*V. bifurcata*	4	99.6%
*V. cavernarum*	3	99.5%
*V. devergens*	16	98.9%
*V. difficilis*	4	100%
*V. foveolata*	12	98.5%
*V. karelica*	4	99.1%
*V. kuusamoensis*	18	99.8%
*V. subdevergens*	3	100%
*V. subjunctiva*	18	98.7%
*V. subtilis*	26	98.7%
*V. vacillans*	10	98.6%

**Figure 3.**
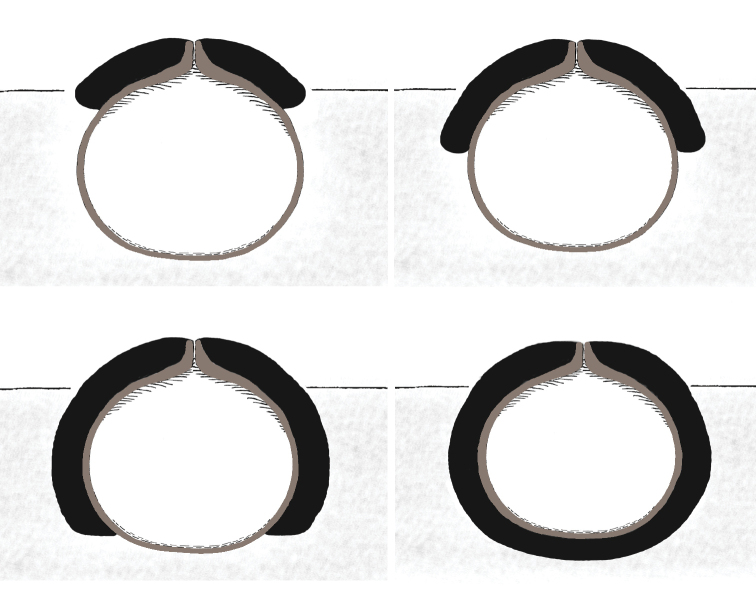
Schematic drawings of sections of perithecia of the study species **A** involucrellum apical **B** involucrellum covering half of the exciple **C** involucrellum reaching the exciple base level **D** involucrellum enveloping the exciple.

Infraspecific morphological variation usually appeared to be rather high. For instance, in most study species, more than one major involucrellum type was detected and infraspecific variation of other perithecium characters was also considerable (Fig. 3, Table 3).

**Table 3.** The main perithecium characters of the study species. Per = Perithecia size (mm), Inv = Involucrellum: ab = absent, ap = apical, ce = covering half of the exciple, bl = to the exciple base level, ee = enveloping the exciple, Invthick = Involucrellum thickness (mm), Exc = Exciple size in diameter (mm), Spores = Ascospore size (mm), minimum, mean and maximum values.

Species	Per	Inv	Invthick	Exc	Spores
*V. bifurcata*	0.13–0.26	ab, ap, bl, ee	0–60	0.18–0.27	21–26–30 × 9–11–13
*V. cavernarum*	0.15–0.28	ap, ce	30–60	0.16–0.32	23–28–34 × 10–12–14
*V. devergens*	0.13–0.40	ab, ap, ce	0–80	0.20–0.35	20–27–35 × 10–13–16
*V. difficilis*	0.18–0.36	ce, bl	40–70	0.16–0.28	23–27–34 × 10–11–13
*V. foveolata*	0.11–0.42	ab, ap, ce	0–60	0.19–0.42	24–30–37 × 10–13–17
*V. fuscozonata*	0.11–0.26	bl	50–60	0.18–0.25	21–26–29 × 10–12–13
*V. karelica*	0.07–0.37	ap, ce	50–70	0.21–0.28	23–28–31 × 10–12–14
*V. kuusamoensis*	0.17–0.45	ce, bl, ee	30–80	0.19–0.29	21–28–34 × 9–12–14
*V. subdevergens*	0.21–0.42	ce, bl, ee	30–80	0.21–0.34	23–28–35 × 11–13–15
*V. subjunctiva*	0.16–0.45	ce, bl, ee	40–100	0.20–0.36	23–30–40 × 12–14–17
*V. subtilis*	0.15–0.44	ap, ce	30–80	0.16–0.33	20–25–31 × 8–10–13
*V. vacillans*	0.15–0.47	ap, ce, bl	30–90	0.15–0.26	18–25–32 × 8–12–15

## Discussion

Based on the Maximum Likelihood analysis, all the studied species in the morphogroup with large spores, perithecia leaving pits in the rock and a pale thin thallus belong to the *Thelidium* group, but they do not form a monophyletic group. Instead, they are widely distributed within the *Thelidium* group.

Molecular data show that the number of the Finnish species in the morphogroup is higher than previously expected. Similar results have often been obtained from other molecular studies in *Verrucaria* (Orange 2013a; Pykälä et al. 2019), as well as in many other lichen groups (e.g. Kraichak et al. 2015; Jüriado et al. 2017; Launis et al. 2019). We could find previously-published names for only four of the species, even though the type material of 47 previously-described European species, potentially belonging to the *Thelidium* group, was studied. This suggests that Fennoscandian and Central European *Verrucaria* mycobiota largely differ from each other. Similar results have been obtained amongst other previously-studied *Verrucaria* taxa (Pykälä et al. 2017a, b, 2019).

Of the new species, *Verrucaria bifurcata*, *V. cavernarum*, *V. difficilis* and *V. subtilis* form a weakly-supported group of closely-related species (*V. subtilis* complex). Similarly, *V. devergens*, *V. karelica* and *V. subdevergens* are closely related and belong to the so-called *V. devergens* complex. The species in both complexes can be seen as examples of cryptic species: while they are genetically distinct, there are no clear morphological differences that can used to separate between different lineages (see Crespo and Lumbsch 2010). However, there seems to be some ecogeographical differences between the species (as discussed in the Taxonomy section). Furthermore, there are differences in the infraspecific morphological variation between the species.

*Verrucaria foveolata* and *V. subjunctiva* do not differ in their ITS sequences, even if the species are usually identifiable, based on morphology. Furthermore, the two species have ecological and geographical differences in Finland, as discussed below. Thus, we prefer to treat them as different species pending further study using other molecular markers. It is generally acknowledged that ITS sometimes fails to separate closely-related species of lichens (see, for instance, Leavitt et al. 2013; Pino-Bodas et al. 2013; Magain and Sérusiaux 2015).

We included multiple specimens per species in our study to examine genetic and morphological infraspecific variation. Interestingly, in most of the species, we found one or a few specimens that differed morphologically from the other specimens and could not be reliably identified at species level. This suggests that a rather high number of specimens needs to be sequenced to cover the infraspecific morphological variation of the species. Even if the studied species are characterised by a high infraspecific morphological variation and even overlap in morphology, the infraspecific variation in the ITS sequence is rather low. This result is similar to the recently analysed *Verrucaria kalenskyi* – *V. xyloxena* complex (Pykälä et al. 2019). The results suggest that reliable identification of the studied species, based on morphology, is often not possible, especially if a specimen lacks well-developed spores. Particularly, specimens with unusually small or large spores or with involucrellum deviating from normal are easily misidentified. The studied species show, on average, differences in several morphological characters, but there is a considerable overlap in all these characters between different species. Such semi-cryptic species may be common in *Verrucaria* and related genera (Orange 2012, 2013a, 2014; Thüs et al. 2015; Pykälä et al. 2017b, 2018, 2019).

For example, the occurrence of dark lines between contiguous conspecific thalli varies between the species. Such lines are common in *V. vacillans*, fairly common in the *V. devergens* complex, infrequent in *V. kuusamoensis* and absent in *V. foveolata*, *V. subjunctiva* and in the *V. subtilis* complex.

The study group is characterised by a predominance of a dark exciple wall. In most species, pale exciple walls have not been seen. Pale exciples are rather common only in *V. subtilis*, although most specimens have only dark exciples. In *V. kuusamoensis*, over 95% of the specimens have only dark exciples, but very few specimens (two confirmed by ITS) include only or also pale exciples.

The occurrence of a halonate perispore has been confirmed for all studied Finnish species, but *V. karelica*. However, many specimens were studied when a few years (3–6 years) old. Then the occurrence of the halonate perispore was often not confirmed. In specimens that are a few years old, a halonate perispore was seen only in few spores or it was not found. It remains to be studied whether a halonate perispore can always be detected in fresh material.

Most study species have a northern distribution in Finland. The result was unexpected, because most previously-described species in the morphogroup are from Central Europe. This result suggests that most Finnish species may be restricted to the boreal and arctic zones or be at least rare south of the boreal zone. Dolomite rocks in the Oulanka area in the biogeographical Province of Koillismaa have the highest species richness of large-spored *Verrucaria* leaving pits in the rock. *Verrucaria subtilis* and *V. bifurcata* may be the only species in the group which seem to be more common in southern than in northern Finland and the latter species may possibly occur only in southern Finland.

## Taxonomy

Species descriptions are based on the Finnish sequenced specimens.

### Verrucaria bifurcata

Taxon classificationFungiVerrucarialesVerrucariaceae

Pykälä, Kantelinen & Myllys sp. nov.

B9D322E1-59CA-5608-830E-E63C4B3CF4AD

835669

Fig. 2A

#### Diagnosis.

Species characterised by pale, usually endolithic thallus, perithecia leaving shallow to deep pits in the rock, very variable involucrellum appressed to the exciple and ascospores (21–)24–28(–30) × (9–)10–12(–13) mm, morphologically rather similar to the other Finnish species of the *V. subtilis* complex, but the ITS sequence divergence between the species is 1.7–3.9%.

#### Holotype.

Finland. Varsinais-Suomi, Länsi-Turunmaa (Parainen), Ersby, 150 m SE of Stormossen, abandoned lime quarry, quarry waste hill, S-slope, on pebbles, 27 m alt., 60°17'N, 22°15'E, 3 Sept 2009 J. Pykälä 36722 (H9205739, GenBank accession number: MT229720).

#### Description.

Prothallus absent. Thallus white, grey or pale greyish-brown, endolithic or thinly epilithic, continuous or small patches surrounding perithecia, ca. 20–60 mm thick, algal cells (4–)5–8 mm. Perithecia 0.13–0.26 mm in diam., (1/2–)3/4(–1)–immersed, leaving shallow to deep pits in the rock, sometimes thinly thalline covered; 80–140 perithecia/cm^2^. Ostiole inconspicuous, tiny, pale or dark, plane or depressed, ca. 20–30 mm wide. Involucrellum absent, apical, to the exciple base level or enveloping the exciple, 20–60 mm thick, appressed to the exciple. Exciple 0.18–0.27 mm in diam., wall dark brown or black, ca. 20–30 mm thick. Periphysoids ca. 25–35 × 1.5–2.5 mm. Asci 60–104 × 22–33 mm, 8-spored. Ascospores 0-septate, (20.6–)23.8–25.8–27.8(–30.3) × (8.7–)10.0–11.0–12.0(–12.9) mm (n = 117), perispore 1–1.5 mm thick.

#### Habitat and distribution.

All finds are from lime quarries or road cuttings of calcareous rocks. The species seems to prefer pebbles and stones in lime quarries. It occurs both in sun-exposed and rather shady habitats. The specimens are from SW and SE Finland. This suggests that *V. bifurcata* has a southern distribution in Finland.

#### Etylomogy.

The epithet refers to the dualistic nature of the involucrellum of the species: absent or short vs. long or enveloping the exciple.

#### Other specimens examined.

Finland. Varsinais-Suomi, Särkisalo, Förby, E of Vähämaankaula, abandoned lime quarry, beneath NW-facing wall, on stone, 7 m alt., 60°05'N, 22°52'E, 23 July 2008, J. Pykälä 33120 (H); Länsi-Turunmaa (Parainen), Simonby, Gropen, abandoned lime quarry, road cutting of calciferous rock, on pebbles, 15 m alt., 60°16'N, 22°13'E, 16 Sept 2009, J. Pykälä 37228 (H); Etelä-Savo, Kerimäki, Ruokojärvi, Pitkäniemi, abandoned lime quarry, on NE-facing wall, 90 m alt., 61°56'N, 29°00'E, 15 Sept 2011, J. Pykälä 45762 (H).

#### Notes.

*Verrucaria bifurcata* is a somewhat puzzling species as it has a very variable involucrellum. Two specimens are characterised by an absent or small involucrellum and two by a deep reaching involucrellum. In the former case, the involucrellum varies within a specimen from absent to apical. In the latter case, the involucrellum extends to the exciple base level or envelopes the exciple. *Verrucaria bifurcata* cannot be identified with certainty without ITS sequencing. Nevertheless, it shows morphological variation differing from the other species in the *V. subtilis* complex. *Verrucaria bifurcata* is the only species in the *V. subtilis* complex in which involucrellum may be absent or enveloping the exciple. In *V. bifurcata*, the involucrellum is always tightly appressed to the exciple and sometimes it is difficult to find out whether the involucrellum is absent or enveloping the exciple. The specimen 45762 was originally identified as *V. adelminienii* Zschacke (Pykälä 2013). However, the type of *V. adelminienii* is not identifiable (Pykälä 2016). Furthermore, the spore size in the original description (Zschacke 1933) is smaller than the spore size in the Finnish specimen.

### Verrucaria cavernarum

Taxon classificationFungiVerrucarialesVerrucariaceae

Pykälä & Myllys sp. nov.

2EF3C19D-1815-53D2-8038-D87CF138CC61

835670

Fig. 2B

#### Diagnosis.

Species morphologically somewhat similar to *V. subtilis*, ascospores slightly larger: (23–)25–30(–34) × (10–)11–13(–14) mm and the ITS sequence divergence between the species is 2.8–3.4%.

#### Holotype.

Finland. Koillismaa, Kuusamo, Oulanka National Park, Mataraniemi, shore of Oulankajoki river, treeless stony river shore, on dolomite stones, 145 m alt., 66°22'N, 29°20'E, 26 Aug 2011, J. Pykälä 45168 (H9205102, GenBank accession number: MT229725).

#### Description.

Prothallus absent. Thallus grey to pale greyish-brown, endolithic or thinly epilithic, continuous, 20–80 mm thick, algal cells 5–8 mm. Perithecia 0.15–0.28 mm in diam., 1/2–1-immersed, leaving shallow to deep pits in the rock, often surrounded by thallus collar, few perithecia thinly thalline covered; 80–160 perithecia/ cm^2^. Ostiole inconspicuous, dark, plane or depressed. Involucrellum apical to covering half of the exciple, in one specimen also few longer involucrella almost reaching the exciple base level present, 30–60 mm thick, appressed to the exciple or slightly diverging from the exciple. Exciple 0.16–0.32 mm, wall dark brown or black, ca. 15–25 mm thick. Periphysoids (25–)30–40(–50) × 1.5–2.5 mm, branching. Asci 8-spored. Ascospores 0-septate, two 1-septate spores seen in one specimen, (23.1–)25.1–27.5–29.8(–34.1) × (9.8–)10.7–11.6–12.6(–13.7) mm (n = 111), perispore 1 mm thick.

#### Habitat and distribution.

Two specimens of the species are from SW Finland and one specimen from NE Finland. The three sequenced specimens are from different kinds of habitats: dolomite stone on river shore (apparently periodically submerged), calcareous rock on seashore (perhaps not submerged) and in a lime quarry on pebbles. The species may prefer more humid (but preferably sun-exposed?) habitats than the other species in the *V. subtilis* complex.

#### Etymology.

The perithecia of the species leave shallow to deep pits in the rock when decayed.

#### Other specimens examined.

Finland. Varsinais-Suomi, Raasepori (Karjaa), Knapsby, Mustio lime quarry, deciduous forest on lime quarry waste, on pebbles, 45 m alt., 60°10'N, 23°49'E, 2 July 2009, J. Pykälä 34527 (H); Länsi-Turunmaa (Iniö), Söderby, Biskopsö island, calcareous rock outcrop on shore of the Baltic Sea, on N-slope, scarce, 7 m alt., 60°20'N, 21°28'E, 9 June 2010, J. Pykälä 37975 (H).

#### Notes.

The species cannot be morphologically separated with certainty from the other species of the *V. subtilis* complex. It is most difficult to separate from *V. subtilis*. On average, *V. cavernarum* has slightly longer (mean 2.3 mm longer than in *V. subtilis*) and broader (mean 1.1 mm broader than in *V. subtilis*) spores and pale exciples have not been found.

### Verrucaria devergens

Taxon classificationFungiVerrucarialesVerrucariaceae

Nyl., Flora 55: 362, 1872 (as V. divergens Nyl., a typographic error)

7C2D5760-1EAC-536C-B9E7-D3105F84B043

#### Type.

[Russia,] Suojärvi, ad saxa calcarea Pöpönsaari, 1870, Norrlin (H!, H-NYL 3036a!, syntypes).

#### Description.

Prothallus absent. Thallus white, grey or pale brown, endolithic, rarely epilithic (two sequenced specimens), thin, continuous, algal cells 5–8 mm, occasionally (three sequenced specimens) contiguous conspecific thalli separated by dark brown lines, 0.13–0.22 mm wide. Perithecia 0.13–0.40 mm in diam., (1/4–)1/2–1-immersed, leaving shallow to deep pits in the rock, few perithecia occasionally not leaving pits, often surrounded by a thalline collar, sometimes thinly thalline covered; 50–140 perithecia/cm^2^. Ostiole usually inconspicuous, pale or dark, plane or depressed, ca. 20–50 mm wide. Involucrellum absent or apical, short, rarely covering half of the exciple (two sequenced specimens), (40–)50–80 mm thick, appressed to the exciple or diverging from the exciple. Exciple 0.20–0.35 mm in diam., wall dark brown or black, ca. 27–40 mm thick, apex thickened to ca. 50–100 mm thick if the involucrellum is absent. Periphysoids ca. 30–50(–60) × 1–2.5 mm, branching or branched-anastomosing. Ascospores 0-septate, (20.2–)24.6–27.4–30.2(–34.8) × (10.2–)11.7–12.6–13.5(–15.7) mm (n = 281), perispore 1 mm thick.

#### Habitat and distribution.

The species is a strict calcicole occurring on calcareous rocks. It may prefer fairly humid habitats. *Verrucaria devergens* seems to be able to tolerate moderate flooding and it also grows on subaquatic calcareous rocks on river shores in the Oulanka area. It is not rare on dolomite rocks in the Oulanka and Kilpisjärvi areas in northern Finland, but seems to be absent from southern Finland.

#### Other specimens examined.

Finland. Koillismaa, Kuusamo, Oulanka National Park, Pikkukönkäänkuru, *Pinus sylvestris*-dominated forest, SW-slope, on dolomite stones, 178 m alt., 66°21'N, 29°19'E, 8 Aug 2009, J. Pykälä 35922 (H); Kuusamo, Oulanka National Park, Pikkukönkäänkuru, dolomite rock crop, on overhanging SW-facing wall, 173 m alt., 66°21'N, 29°19'E, 8 Aug 2009, J. Pykälä 35933 (H); Kuusamo, Oulanka National Park, Pikkuköngäs, N shore of river Oulankajoki, dolomite rock outcrop, on SW-facing wall, 160 m alt., 66°22'N, 29°19'E, 12 Aug 2009, J. Pykälä 36220 (H), 36244 (H), 36245 (H); Kuusamo, Oulanka National Park, Pikkuköngäs, N shore of river Oulankajoki, dolomite rock outcrop, stony shore, on stones, 160 m alt., 66°22'N, 29°19'E, 12 Aug 2009, J. Pykälä 36234 (H); Kuusamo, Oulanka National Park, Pikkuköngäs, N shore of river Oulankajoki, dolomite rock outcrop, on 1 m high SW-facing wall, 160 m alt. 66°22'N, 29°19'E, 12.VIII.2009, J. Pykälä 36271 (H); Kuusamo, Oulanka National Park, Kiutaköngäs, N shore of river Oulankajoki, dolomite rock outcrop, on SE-slope, 150 m alt., 66°22'N, 29°20'E, 12 Aug 2009, J. Pykälä 36304 (H); Kuusamo, Oulanka National Park, Kiutaköngäs, by the rapids, S shore of Oulankajoki river, calciferous (dolomite) schistose rock outcrop, NE-slope, on E-facing wall, 152 m alt., 66°22'N, 29°19'E, 13 Aug 2010, J. Pykälä 39898 (H); Kuusamo, Oulanka National Park, Kiutaköngäs, by the rapids, S shore of Oulankajoki river, calciferous (dolomite) schistose rock outcrop, on gentle NE-slope, 152 m alt., 66°22'N, 29°19'E, 13 Aug 2010, J. Pykälä 39901 (H); Kuusamo, Oulanka National Park, Taivalköngäs, shore of Oulankajoki river, stony river shore, on dolomite stone, 170 m alt., 66°24'N, 29°11'E, 25 Aug 2011, J. Pykälä 45090 (H); Kuusamo, Oulanka National Park, Mataraniemi W, shore of Oulankajoki river, small dolomite rock outcrop, on 40 cm high SE-facing wall, 145 m alt., 66°22'N, 29°20'E, 28 Aug 2011, J. Pykälä 45367 (H); Salla, Oulanka National Park, 400 m N of Savilampi, shore of river Savinajoki, cliff, dolomite rock outcrop, on overhanging NE-facing wall, 177 m alt. 66°25'N, 29°10'E, 13 Aug 2009, J. Pykälä 36344 (H); Salla, Oulanka National Park, Savilampi 1.4 km NE, shore of Savinajoki river, dolomite rock outcrop, SE-slope, on dolomite boulder, 184 m alt., 66°26'N, 29°11'E, 23 Aug 2011, J. Pykälä 44914 (H); Enontekiön Lappi, Enontekiö, Porojärvet, Toskalharji, Toskaljärvi N, fell, gentle SW-slope, dolomite scree, on dolomite boulders , 710 m alt., 69°11'N, 21°26'E, 3 Aug 2011, J. Pykälä 43421 (H); Enontekiö, Kilpisjärvi, Saana, nature reserve, E-part, fell, dolomite rock outcrop, on SW-facing wall, 880 m alt., 69°02'N, 20°50'E, 10 Aug 2011, J. Pykälä 44042 (H).

#### Notes.

Based on the ITS phylogeny, *V. devergens*, *V. karelica* and *V. subdevergens* are very closely related. They are here considered as distinct species, based on the ITS phylogeny and because of a barcoding gap between the species. *Verrucaria devergens* is morphologically more variable than previously known (Pykälä 2007). Usually, the species has no involucrellum, but the apex of the exciple is thickened. However, specimens with an apical involucrellum, as well as two specimens in which the involucrellum covers half of the exciple, have an identical ITS sequence compared to the typical *V. devergens*. Typically, *V. devergens* has perithecia varying from half-immersed to immersed in the same specimen, but in some specimens, the perithecia are 3/4–1-immersed, while in a few others, they are 1/4–1/2-immersed.

*Verrucaria devergens* is difficult to separate from *V. foveolata*, *V. karelica* and *V. subdevergens*. *V. devergens* and *V. foveolata* show similar variation in the involucrellum, i.e. absent or apical. *Verrucaria foveolata* has larger spores, but there is a wide overlap in the spore size. *Verrucaria foveolata* usually has immersed perithecia, while *V. devergens* has 1/2–1-immersed perithecia. However, some specimens of *V. devergens* are similar to *V. foveolata* in having 3/4–1-immersed perithecia. No consistent morphological differences were found between *V. devergens* and *V. karelica*, although all specimens of *V. karelica* have an involucrellum. *Verrucaria karelica* may have more often an epilithic thallus and dark lines between contiguous conspecific thalli. *Verrucaria subdevergens* has a longer involucrellum than *V. devergens* in all studied specimens predominantly exceeding half of the exciple.

Specimens of *V. devergens* with untypically deep reaching involucrellum may be difficult to separate from *V. kuusamoensis* and *V. subtilis*. *Verrucaria kuusamoensis* tend to have a smaller exciple and shorter periphysoids, the thallus is usually epilithic and the involucrellum usually exceeds half of the exciple. *Verrucaria subtilis* has thinner and smaller exciple and, on average, smaller spores. In some specimens of *V. subtilis*, pale exciples are present, while they have never been found from *V. devergens*.

### Verrucaria difficilis

Taxon classificationFungiVerrucarialesVerrucariaceae

Pykälä & Myllys sp. nov.

B8281418-3C08-5DD8-A55A-026F22E59110

835671

Fig. 2C

#### Diagnosis.

Species characterised by perithecia 1/4–3/4-immersed, leaving usually shallow pits, involucrellum covering half of the exciple or almost to the exciple base, ascospores (23–)25–29(–34) × (10–)11–12(–13) mm, morphologically rather similar to the other Finnish species of the *V. subtilis* complex, but the sequence divergence in ITS 1.7–2.6%.

#### Holotype.

Finland, Varsinais-Suomi, Karkkila, Haavisto, 100 m S of E-part of Iitalammi, S-slope, clear cut herb-rich forest, on calcareous stone, 60°31'N, 24°23'E, 123 m alt., 7 June 2008 J. Pykälä 32687 (H9205096, GenBank accession number: MT229742).

#### Description.

Prothallus absent. Thallus white or grey, inconspicuous, endolithic to thinly epilithic, continuous to irregularly rimose, ca. 20–80 mm thick, algal cells 5–7(–8) mm. Perithecia 0.18–0.36 mm in diam., 1/4–3/4(–1)–immersed, leaving shallow to more rarely deep pits in the rock, often thinly thalline covered except apex; 60–160 perithecia/cm^2^. Ostiole inconspicuous, tiny, pale to usually dark, plane or depressed, ca. 20–30 mm wide. Involucrellum covering half of the exciple or almost to the exciple base, 40–70 mm thick, appressed to the exciple or slightly or moderately diverging from it. Exciple 0.16–0.28 mm in diam., wall dark brown, ca. 20–25 mm thick. Periphysoids (20–)25–35(–40) × 1.5–2.5 mm, some branching. Asci 77–101 × 23–28 mm, 8-spored. Ascospores (22.7–)25.1–27.0–28.9(–33.6) × (9.6–)10.6–11.4–12.3(–13.3) (n = 78), perispore 1 mm thick.

#### Habitat and distribution.

Four sequenced specimens occur: two from SW Finland and two from NE Finland. The species grows on calcareous rocks and in lime quarries, on walls, boulders, stones and pebbles. *Verrucaria difficilis* may prefer half-shady habitats. The species is rare, but may also have been overlooked due to its morphological similarity to several other species.

#### Etymology.

The species may be mixed up with several other species of *Verrucaria*.

#### Other specimens examined.

Finland, Koillismaa, Kuusamo, Oulanka National Park, Kiutaköngäs 400 m N, *Pinus sylvestris*-dominated forest, small dolomite rock outcrop, SW-slope, on pebbles, 165 m alt., 66°22'N, 29°19'E, 2 Aug 2010, J. Pykälä 39060 (H); Kuusamo, Juuma, Niskakoski, calciferous (dolomite) schistose rock outcrop, on calciferous boulder, 225 m alt., 66°13'N, 29°24'E, 22 Aug 2011, J. Pykälä 44811 (H); Uusimaa, Vantaa, Sotunki, Bisa, 300 m E-NE, herb-rich forest, abandoned lime quarry, on SW-facing wall, 35 m alt., 60°17'N, 25°08'E, 7 June 2011, J. Pykälä 41859 (H).

#### Notes.

Based on the ITS phylogeny, *V. difficilis* belongs to the *V. subtilis* complex with *V. bifurcata*, *V. cavernarum* and *V. subtilis*. The involucrellum is usually longer than in *V. cavernarum* and *V. subtilis*. Furthermore, the perithecia of *V. difficilis* are, on average, less immersed, often only 1/4–1/2-immersed in rock. *Verrucaria bifurcata* differs in more immersed perithecia with the involucrellum appressed to the exciple. Nevertheless, *V. difficilis* may not be identified with certainty without sequencing. *Verrucaria difficilis* is also difficult to separate from *V. kuusamoensis*. This species has slightly longer spores and the perithecia commonly leave deep pits in the rock.

A Genbank sequence *Verrucaria calkinsiana* Servít (KT695332) has 98% similarity to *V. difficilis* and it remains to be studied whether it is a closely-related species or possibly conspecific. Based on the morphology of the type specimen (PRM-857016!), *V. calkinsiana* does not belong to the *V. subtilis* complex and the sequenced specimen is apparently misidentified.

### Verrucaria foveolata

Taxon classificationFungiVerrucarialesVerrucariaceae

(Flörke) A. Massal., Ric. auton. lich. crost.: 346, 1852

73BB098F-0BA9-5568-B221-5EC3059E0934

= Verrucaria latzeliana Servít, Stud. Bot. Čech. 9: 89, 1948. Type. Ragusa: Gartenmauer am 3. Aquidotto, ca. 200 m, Kalk, 28.7.1907, A. Latzel (PRM-859178!, holotype)
Verrucaria schraderi Sommerf. var. foveolata Flörke, Deutschl. Lich. 6, 1815. Basionym.

#### Type.

Not seen. Protologue: “auf Kalksteinen bei Rüdersdorf”.

#### Description.

Prothallus absent. Thallus white, grey or pale brown, endolithic, often inconspicuous, rarely thinly epilithic, algal cells 5–9 mm. Perithecia 0.11–0.42 mm, (1/2–)3/4–1-immersed in rock, leaving deep pits in the rock, commonly surrounded by a thallus collar, sometimes covered by a thin thalline layer except for the apex; (30–)60–120 perithecia/cm^2^. Ostiole usually inconspicuous, tiny, pale or dark, plane or depressed, ca. 20–40(–50) mm wide, wider ostiolar depression rarely present up to 80 mm wide. Involucrellum absent or apical, rarely covering half of the exciple, 40–60 mm thick. Exciple 0.19–0.42 mm in diam., usually round, but sometimes pear-shaped or at least longer than broad, medium brown (rarely), dark brown or black, ca. (20–)25–43(–60) mm thick, apex sometimes thickened to ca. 40–60 mm thick if the involucrellum is absent. Periphysoids ca. (30–)40–65 × 1–2(–3) mm, branching. Asci 78–102 × 27–39 mm, 8-spored. Ascospores 0-septate, rarely solitary spores 1-septate, (23.6–)27.4–30.5–33.7(–37.3) × (10.4–)12.1–13.4–14.6(–17.1) mm (n = 197), perispore 1–1.5 mm thick.

#### Habitat and distribution.

The species grows on calcareous rocks and in lime quarries, both on sun-exposed and shady rocks, both in southern and in northern Finland.

#### Other specimens examined.

Finland. Varsinais-Suomi, Lohja, Torhola, 400 m E of Torhola cave, S-slope, calcareous rock outcrop, 40 m alt., 60°15'N, 23°52'E, 20 July 2007, J. Pykälä 31528 (H); Salo (Kisko), Leilä, Kalkuuni, *Pinus sylvestris*-dominated forest, SW-slope, on calcareous rock wall, 60 m alt., 60°12'N, 23°35'E, 14 July 2009, J. Pykälä 34953 (H); Länsi-Turunmaa (Korppoo), Åfvensår, Kilamo, abandoned lime quarry, on SW-facing wall, 13 m alt., 60°17'N, 21°32'E, 28 July 2009, J. Pykälä 35395 (H); Salo (Kisko), Haapaniemi, Plantmaannokka, calcareous rock outcrop on shore of Lake Määrjärvi, on NE-facing wall, 42 m alt. , 60°12'N, 23°31'E, 4 June 2010, J. Pykälä 37728 (H); Salo (Kisko), Jyly, 200 m NE of Purslammi, calcareous rock outcrop, on NW-facing wall, 67 m alt., 60°14'N, 23°36'E, 17 June 2010, J. Pykälä 38119 (H); Kemiönsaari (Dragsfjärd), Olmos, Kolaskär island, calcareous rock outcrop on shore of the Baltic Sea, beneath SE-facing wall, on pebbles, 2 m alt., 60°03'N, 22°19'E, 12 July 2010, J. Pykälä 38719 (H); Koillismaa, Kuusamo, Oulanka, Putaanoja, 500 m W-NW of Hautala, *Pinus sylvestris*-dominated semi-open forest, dolomite rock outcrop, on N-slope, 230 m alt., 66°22'N, 29°25'E, 9 Aug 2009, J. Pykälä 35965 (H); Kuusamo, Kallunki, Merenvaara, *Pinus sylvestris*-dominated forest, NW-slope, small dolomite rock outcrop, on W-facing wall, 225 m alt., 66°20'N, 29°20'E, 2 Aug 2010, J. Pykälä 39028 (H); Kuusamo, Oulanka National Park, Kiutaköngäs 400 m N, SE-slope, *Pinus sylvestris*-dominated forest, small dolomite rock outcrop, on SW-facing wall, 170 m alt., 66°22'N, 29°19'E, 5 Aug 2010, J. Pykälä 39294 (H); Salla, Oulanka National Park, W of Savikoski, cliff, dolomite rock outcrop, NE-slope, on dolomite boulder, 185 m alt., 66°25'N, 29°10'E, 17 Aug 2010, J. Pykälä 40195 (H); Kuusamo, Oulanka National Park, Taivalköngäs, shore of Oulankajoki river, *Picea abies*-dominated herb-rich forest, dolomite rock outcrop, NE-slope, on dolomite boulder, 174 m alt., 66°24'N, 29°11'E, 20 Aug 2011, J. Pykälä 44553 (H); Salla, Hautajärvi, Kurtinniittykuru, dolomite rock outcrop, on flat surface, 195 m alt., 66°26'N, 29°09'E, 24 Aug 2011, J. Pykälä 44952 (H).

#### Notes.

Fennoscandian specimens of *Verrucaria* with large spores, perithecia leaving deep pits in the rock and immersed in rock, lacking an involucrellum and with endolithic pale thallus have been predominantly treated as *V. foveolata* (e.g. Foucard 2001). It remains uncertain whether *V. foveolata* is the correct name for this common species, as the type material was not located. The absence of involucrellum has been used as the main character to separate *V. foveolata* from morphologically-similar species with apical involucrella, such as *V. dolomitica* (A. Massal.) Kremp. (Breuss 2004). However, the sequenced Finnish specimens with an apical involucrellum do not differ from specimens without an involucrellum.

Based on the ITS phylogeny, *Verrucaria foveolata* and *V. subjunctiva* are not monophyletic, but together form a strongly-supported group. However, the two taxa are, for the time being, treated as separate species pending further study. Most specimens can be identified by their morphology, although we found some intermediate specimens having morphological characters pointing to both species. However, overlap in the morphology is not larger than compared to several, not closely related species of *Verrucaria*. *Verrucaria foveolata* is more difficult to be separated from *V. devergens* (see the species) than from *V. subjunctiva*. Furthermore, some ecological and biogeographical differences seem to occur between *V. foveolata* and *V. subjunctiva*. *Verrucaria subjunctiva* has not been found from lime quarries, whereas several populations of *V. foveolata* occur in lime quarries. *Verrucaria foveolata* is fairly common on calcareous rocks both in southern and northern Finland, whereas *V. subjunctiva* is rare in southern Finland.

### Verrucaria fuscozonata

Taxon classificationFungiVerrucarialesVerrucariaceae

Pykälä, Kantelinen & Myllys sp. nov.

665AE0D5-9FF2-5765-9D6E-7AF9766124AA

835672

Fig. 2D

#### Diagnosis.

Species characterised by dark lines between contiguous conspecific thalli, pale endolithic thallus, small perithecia leaving shallow to deep pits in the rock, involucrellum reaching the exciple base level and appressed to the exciple, ascospores measuring (21–)24–28(–29) × (10–)11–12(–13) mm.

#### Holotype.

Finland. Koillismaa, Kuusamo, Oulanka National Park, Pikkuköngäs, N shore of river Oulankajoki, dolomite rock outcrop, on SW-facing wall, 160 m alt., 66°22'N, 29°19'E, 12 Aug 2009, J. Pykälä 36222 (H, GenBank accession number: MT229758).

#### Description.

Prothallus not seen. Thallus pale grey, endolithic, dark lines between contiguous conspecific thalli, 0.21–0.35 mm wide. Perithecia 0.11–0.26 mm in diam., (1/2–)3/4–immersed, leaving shallow to deep pits in the rock, surrounded by a thallus collar; 120–140 perithecia/cm^2^. Ostiole inconspicuous, tiny, pale to dark, plane or depressed, ca. 30 mm wide. Involucrellum reaching the exciple base, 50–60 mm thick, appressed to the exciple. Exciple 0.18–0.25 mm in diam., wall dark brown to black. Periphysoids ca. 25–35 × 2–2.5 mm. Asci 8-spored. Ascospores 0-septate, (21.2–)24.5–26.5–28.4(–29.4) × (10.0–)10.9–11.7–12.5(–13.2) mm (n = 36), perispore 1 mm thick.

#### Habitat and distribution.

The only known specimen is from a dolomite rock on a river shore in north-eastern Finland, in Kuusamo.

#### Etymology.

The only specimen available is characterised by dark lines between contiguous conspecific thalli.

#### Notes.

*Verrucaria fuscozonata* did not group with any of the examined species in the ITS phylogeny. However, it is morphologically rather similar to *V. bifurcata*, *V. kuusamoensis* and *V. subdevergens*. In *V. bifurcata*, dark lines between contiguous conspecific thalli are absent and the involucrellum usually thinner. In *V. kuusamoensis* and *V. subdevergens*, the spores are larger and the perithecia occur less densely. More material is needed to find out whether *V. fuscozonata* can be unambiguously identified, based on morphology only.

### Verrucaria karelica

Taxon classificationFungiVerrucarialesVerrucariaceae

Vain., Acta Soc. Fauna Flora Fenn. 49(2): 46, 1921

E37C0885-451E-51EB-9E99-F6A59705CE12

#### Type.

Russia, Karelia Onegensis, Mundjärvi, supra saxa dolomitica cinerea, J. P. Norrlin (H-NYL 3146!, H!, syntypes).

#### Description.

Prothallus absent. Thallus white, grey or pale greyish-brown, endolithic or thinly epilithic, farinose, algal cells 5–8 mm, contiguous conspecific thalli often separated by dark lines, 0.13–0.22 mm wide. Perithecia 0.07–0.37 mm, (1/2–)3/4–1-immersed, leaving shallow to usually deep pits in the rock, surrounded by a thalline collar; 40–80 perithecia/cm^2^. Ostiole pale or dark, plane or depressed ca. 20–40(–60) mm wide. Involucrellum apical or covering half of the exciple, 50–70 mm thick, appressed to the exciple or diverging from the exciple. Exciple 0.21–0.28 mm in diam., wall dark brown to black, ca. 20–31 mm thick. Periphysoids ca. 30–50 × 2–2.5(–3) mm. Asci ca. 66–84 × 26–33 mm, 8-spored. Ascospores 0-septate, (23.2–)26.2–27.9–29.5(–31.3) × (10.3–)11.7–12.3–13.0(–14.1) mm (n = 63), perispore not seen, but may have vanished during storage.

#### Habitat and distribution.

This species is known from Finland only from the Oulanka area in the biogeographical province of Koillismaa in NE Finland where it grows on dolomite rocks. It seems to occur in fairly shady habitats.

#### Other specimens examined.

Finland. Koillismaa, Salla, Oulanka National Park, Savikoski 300 m W, *Pinus sylvestris*-forest, steep N-slope, dolomite rock outcrop, on N-facing wall, 180 m alt., 66°25'N, 29°10'E, 10 Aug 2010, J. Pykälä 39625 (H); Kuusamo, Oulanka, Putaanoja, 500 m W-NW of Hautala, NE-slope, dolomite rock outcrop, on 50 cm high SW-facing wall, scarce, 232 m alt., 66°22'N, 29°25'E, 15 Aug 2010, J. Pykälä 39991 (H); Kuusamo, Oulanka National Park, Kiutaköngäs N, steep S-slope, *Pinus sylvestris*-dominated forest, dolomite rock outcrop, on SW-facing wall, 182 m alt., 66°22'N, 29°19'E, 19 Aug 2010, J. Pykälä 40325 (H); Salla, Oulanka National Park, W of Savikoski, cliff, dolomite rock outcrop, on NE-facing wall, scarce, 185 m alt., 66°25'N, 29°10'E, 17 Aug 2010, J. Pykälä 40235 (H).

#### Notes.

The type specimens of *V. karelica* have epilithic thalli and contiguous conspecific thalli are separated by dark lines. None of the Finnish specimens has both epilithic thalli and dark lines. However, one of the sequenced specimens has epilithic thalli and another specimen has dark lines. Thus, based on morphology, this entity probably belongs to *V. karelica*. The type locality of *V. karelica* (Vainio 1921) is situated rather close to the Oulanka area, suggesting that the species would probably occur in the Oulanka area. The species is closely related to *V. devergens* and *V. subdevergens. V. devergens* and *V. karelica* may not be unambiguously separated by morphology only. *Verrucaria devergens* usually has endolithic thalli and several specimens lack an involucrellum. *Verrucaria karelica* may be absent from subaquatic habitats unlike *V. devergens* which often grows on river shores. *Verrucaria subdevergens* has an involucrellum usually exceeding half of the exciple height. The species is also difficult to be separated from several other species of *Verrucaria* belonging to the *Thelidium* group. *Verrucaria cavernarum*, *V. difficilis* and *V. subtilis* always lack dark lines between contiguous conspecific thalli and the spores are smaller. *Verrucaria kuusamoensis* usually has an involucrellum exceeding half of the exciple.

### Verrucaria kuusamoensis

Taxon classificationFungiVerrucarialesVerrucariaceae

Pykälä, Kantelinen & Myllys sp. nov.

AA67772A-9E6F-5888-8E89-F1323CA6EC3C

835673

Fig. 2E

#### Diagnosis.

Species characterised by pale, usually thinly epilithic thallus, rather large perithecia leaving shallow to deep pits in the rock, involucrellum usually covering more than half of the exciple, ascospores (21–)26–30(–34) × (9–)11–13(–14) mm, morphologically difficult to separate from *V. subdevergens*, but the sequence divergence in ITS 6.8–7.4%.

#### Type.

Finland. Koillismaa, Kuusamo, Juuma, Oulanka National Park, Hautaniitynvuoma, gorge, dolomite rock outcrop, on high NE-facing wall, 190 m alt., 66°15'N, 29°26'E, 21 Aug 2011, J. Pykälä 44703 (H9205113 – holotype, UPS – isotype, GenBank accession number: MT229776).

#### Description.

Prothallus absent. Thallus white, grey or more rarely pale brown, endolithic or usually thinly epilithic, continuous or rimose, often farinose, up to 0.2 mm thick, algal cells (4–)5–7 mm, contiguous conspecific thalli sometimes separated by a dark line, 0.12–0.35 mm wide, present in only few specimens. Perithecia 0.17–0.45 mm in diam., (1/4–)1/2–3/4(–1)-immersed, leaving shallow to deep pits in the rock, rarely few perithecia not leaving pits, often thinly thalline covered except apex; (30–)40–120 perithecia/cm^2^. Ostiole tiny, pale or dark, plane or depressed, ca. 20–40(–60) mm wide, occasionally wider ostiolar depression up to 110 mm wide. Involucrellum covering half of the exciple or to the exciple base level, rarely in few perithecia enveloping the exciple, (30–)40–70(–80) mm thick, appressed to the exciple or slightly or moderately diverging from it. Exciple 0.19–0.29 mm in diam., wall dark brown or black, rarely pale, ca. 20–42 mm thick. Periphysoids ca. (20–)25–40 × (1.5–)2–2.5(–3) mm. Asci 68–102 × 25–34 mm, 8-spored. Ascospores 0-septate, (21.4–)25.5–27.9–30.3(–34.5) × (9.3–)11.3–12.2–13.1(–14.2) mm (n = 312), perispore 1 mm thick.

#### Habitat and distribution.

*Verrucaria kuusamoensis* is rather common on dolomite rocks in the Oulanka area in the municipalities of Kuusamo and Salla in the biogeographical Province of Koillismaa (Ks). It seems not to occur in southern Finland.

#### Etymology.

Most specimens of the species originate from the Kuusamo area.

#### Other specimens examined.

Finland. Koillismaa, Kuusamo, Paljakka, E shore of Kuusinkijoki river, Kiukaankorva, dolomite rock outcrop, on overhanging NW-facing wall, scarce, 213 m alt., 66°11'N, 29°38'E, 5 Aug 2009, J. Pykälä 35710 (H); Kuusamo, Oulanka National Park, Pikkukönkäänkuru, herb-rich heath forest, small dolomite rock outcrop, on W-facing wall, 165 m alt., 66°21'N, 29°19'E, 6 Aug 2009, J. Pykälä 35857 (H); Kuusamo, Oulanka National Park, Pikkukönkäänkuru, dolomite rock outcrop, on SW-facing wall, 175 m alt., 66°21'N, 29°19'E, 8 Aug 2009, J. Pykälä 35920 (H); Kuusamo, Oulanka National Park, Pikkuköngäs, N shore of river Oulankajoki, dolomite rock outcrop, on SW-facing wall, 160 m alt., 66°22'N, 29°19'E, 12 Aug 2009, J. Pykälä 36254 (H); Kuusamo, Oulanka National Park, Kiutaköngäs, *Pinus sylvestris*-dominated forest, steep SE-slope, dolomite rock outcrop, on SE-facing wall, rather scarce, 175 m alt., 66°22'N, 29°19'E, 12 Aug 2009, J. Pykälä 36294 (H); Salla, Oulanka National Park, 400 m N of Savilampi, shore of river Savinajoki, dolomite rock outcrop, on NE-slope, scarce, 178 m alt., 66°25'N, 29°10'E, 13 Aug 2009, J. Pykälä 36335 (H); Kuusamo, Oulanka National Park, Kiutaköngäs 400 m N, *Pinus sylvestris*-dominated forest, small dolomite rock outcrop, on SW-slope, 165 m alt., 66°22'N, 29°19'E, 2 Aug 2010, J. Pykälä 39052 (H); Kuusamo, Oulanka National Park, Kiutaköngäs, by the rapids, S shore of Oulankajoki river, calciferous (dolomite) schistose rock outcrop, NE-slope, on E-facing wall, rather scarce, 152 m alt., 66°22'N, 29°20'E, 13 Aug 2010, J. Pykälä 39900 (H); Salla, Oulanka National Park, W of Savikoski, cliff, dolomite rock outcrop, on NW-facing wall, very scarce, 185 m alt., 66°25'N, 29°10'E, 17 Aug 2010, J. Pykälä 40219 (H); Kuusamo, Oulanka National Park, Taivalköngäs, shore of Oulankajoki river, *Picea abies*-dominated herb-rich forest, dolomite rock outcrop, on NE-facing wall, 175 m alt., 66°24'N, 29°11'E, 20 Aug 2011, J. Pykälä 44563 (H); Kuusamo, Oulanka National Park, Taivalköngäs, shore of Oulankajoki river, *Picea abies*-dominated herb-rich forest, dolomite rock outcrop, on E-facing wall, 175 m alt., 66°24'N, 29°11'E, 20 Aug 2011, J. Pykälä 44570 (H); Kuusamo, Juuma, Oulanka National Park, Hautaniitynvuoma, gorge, dolomite rock outcrop, on high NE-facing wall, 190 m alt., 66°15'N, 29°26'E, 21 Aug 2011, J. Pykälä 44694 (H), 44696 (H); Kuusamo, Juuma, Oulanka National Park, Hautaniitynvuoma, gorge, stony NW-slope with sparse stunted birches, close to bottom, on dolomite stone, 182 m alt., 66°15'N, 29°26'E, 21 Aug 2011, J. Pykälä 44744 (H); Salla, Hautajärvi, Kurtinniittykuru, cliff, dolomite rock outcrop, on SE-facing wall, scarce, 195 m alt., 66°26'N, 29°09'E, 24 Aug 2011, J. Pykälä 44980 (H); Kuusamo, Oulanka National Park, Halosenkuru gorge, NW-slope, *Picea abies*-dominated forest, dolomite rock outcrop, on NW-facing wall, 215 m alt., 66°21'N, 29°26'E, 27 Aug 2011, J. Pykälä 45231 (H); Kuusamo, Oulanka National Park, Halosenkuru, gorge, dolomite rock outcrop, on SE-facing wall, scarce, 235 m alt., 66°21'N, 29°26'E, 28 Aug 2011, J. Pykälä 45330 (H).

#### Notes.

The exciple wall of *V. kuusamoensis* is usually dark brown or black. However, one specimen with a pale exciple wall and one specimen with both pale and dark exciple walls have similar ITS sequences compared to the specimens with dark exciple walls. This species was erroneously reported as *V. subjunctiva* by Pykälä (2010a), while *V. subjunctiva* has larger spores and longer periphysoids. *Verrucaria devergens* has a shorter involucrellum. *Verrucaria kuusamoensis* may be most difficult to separate from *V. subdevergens* and *V. difficilis*. These species show wide overlap in their morphology. *Verrucaria subdevergens* more often has pale brownish thallus and slightly longer periphysoids. *Verrucaria difficilis* has, on average, less-developed thallus and the perithecia and the spores slightly smaller.

### Verrucaria subdevergens

Taxon classificationFungiVerrucarialesVerrucariaceae

Pykälä & Myllys sp. nov.

3E7604DB-240A-58B1-AFCB-34D8FFF44FF3

835674

Fig. 2F

#### Diagnosis.

Differing from *V. devergens* by longer involucrellum, morphologically difficult to separate from *V. kuusamoensis*, but the sequence divergence in ITS 5.4–6.0%.

#### Holotype.

Finland, Koillismaa, Kuusamo, Oulanka National Park, Taivalköngäs, shore of Oulankajoki river, dolomite rock outcrop, on gentle NE-slope, 165 m alt., 66°24'N, 29°11'E, 25 Aug 2011, J. Pykälä 45109 (holotype: H9205097, GenBank accession number: MT229783).

#### Description.

Prothallus absent. Thallus white, grey, ochraceous or pale greyish-brown, endolithic to thinly epilithic, continuous to irregularly rimose, in one specimen contiguous conspecific thalli separated by a dark line. Perithecia 0.21–0.42 mm, 1/2–3/4-immersed, leaving shallow to deep pits in the rock, often surrounded by a thallus collar, in one specimen, thalline covered except apex, thalline cover 8–20 mm thick; 80–120 perithecia/cm^2^. Ostiole inconspicuous, tiny, pale to dark, plane or depressed, ca. 20–40 mm wide. Involucrellum covering half of the exciple or to the exciple base, in few perithecia may envelope the exciple, 30–80 mm thick, in one specimen, often apically thickened to 50–70 mm thick, appressed to the exciple. Exciple 0.21–0.34 mm in diam., wall blackish-brown, ca. 15–25 mm thick. Periphysoids ca. 25–50 × 1.5–2 mm. Asci 82–94 × 27–33 mm, 8-spored. Ascospores 0-septate, (23.0–)25.4–28.2–31.0(–34.9) × (11.2–)12.0–13.0–13.9(–15.2) mm (n = 83), perispore 1–1.5 mm thick.

#### Habitat and distribution.

All three finds are from the Oulanka area in NE Finland where the species grows on dolomite rock outcrops and on a dolomite boulder.

#### Etymology.

The species is close to *V. devergens*.

#### Other specimens examined.

Finland. Koillismaa, Kuusamo, Oulanka National Park, Kiutaköngäs 400 m N, *Pinus sylvestris*-herb-rich forest, small dolomite rock outcrop, on small S-facing wall, 165 m alt., 66°22'N, 29°19'E, 3 Aug 2010, J. Pykälä 39128 (H); Kuusamo, Oulanka National Park, Taivalköngäs, shore of Oulankajoki river, *Picea abies*-dominated herb-rich forest, dolomite rock outcrop, NE-slope, on dolomite boulder, 174 m alt., 66°24'N, 29°11'E, 20 Aug 2011, J. Pykälä 44550 (H).

#### Notes.

This species is close to *V. devergens* and *V. karelica*, based on the ITS phylogeny. It differs from these species in a longer involucrellum mainly exceeding half of the exciple. Morphologically, *V. subdevergens* is most difficult to separate from *V. kuusamoensis*, which tends to have shorter periphysoids and the thallus is more often white.

### Verrucaria subjunctiva

Taxon classificationFungiVerrucarialesVerrucariaceae

Nyl., Flora 67: 218, 1884

14EE11C9-D797-5D61-A968-4CBEE29E1323

392473

= ?Verrucaria lacerata Servít, Stud. Bot. Čech. 11: 115, 1950. Type. Slovakia, Tatry Bielské, rup, calc. pr. Tatranská kotlina, 800 m alt., 1925 Suza (PRM-859169!, syntype)

#### Type.

[Russia,] Sibiria Septentrionalis: Si nus Konyam ad fretum Bering, 64°50' lat. bor., 173° long. occid. (Greenw.) 28–30.7.1879 E. Almquist (S-L46!, lectotype, designated here); Fretum Behring, Kongar Bay, E. Almquist (H-NYL 3512!, isolectotype).

#### Description.

Prothallus absent. Thallus white or grey, rarely pale ochraceous, endolithic or thinly epilithic, continuous or rimose, up to 0.1 mm thick, algal cells 5–8 mm. Perithecia (0.16–)0.23–0.45 mm in diam., (1/4–)1/2–3/4(–1)-immersed, not leaving pits or usually leaving shallow or deep pits in the rock, sometimes covered by a thin thalline layer except for the apex, often surrounded by a thalline collar; ca. (10–)30–100(–120) perithecia/cm^2^. Ostiole tiny, pale or dark, plane or depressed, ca. 20–40(–50) mm wide, ostiolar depression rarely wide, up to 130 mm wide. Involucrellum exceeding half of the exciple or reaching the exciple base level, rarely enveloping the exciple, (40–)50–100 mm thick, appressed to the exciple or slightly to moderately diverging from the exciple. Exciple 0.20–0.36 mm in diam., wall dark brown or black, ca. 22–45 mm thick. Periphysoids ca. 30–60 × (1–)1.5–2.5 mm, branching. Asci 84–109 × 32–40 mm, 8-spored. Ascospores 0-septate, rarely very few spores 1-septate, (23.4–)27.0–30.4–33.8(–40.1) × (11.7–)12.6–13.8–15.0(–17.4) mm (n = 242), perispore 1–2 mm thick.

#### Habitat and distribution.

The species occurs on calcareous rocks in both sun-exposed and shady sites. Most sequenced specimens are from the biogeographical province of Koillismaa. Three sequenced specimens (two localities) originate from eastern Finland (biogeographical Province of Pohjois-Karjala) and three (two localities) from southern Finland (biogeographical Province of Varsinais-Suomi). In southern Finland, the species seems to be very rare. *Verrucaria subjunctiva* has not been collected in Finland from lime quarries.

#### Other specimens examined.

Finland. Varsinais-Suomi, Länsi-Turunmaa (Korppoo), Åfvensår, Kilamo, calcareous rock outcrop, on flat rock, scarce, 17 m. alt., 60°17'N, 21°32'E, 28 July 2009, J. Pykälä 35326 (H); Länsi-Turunmaa (Korppoo), Åfvensår, Kilamo, calcareous rock outcrop, on flat rock, on pebbles, 17 m alt., 60°17'N, 21°32'E, 28 July 2009, J. Pykälä 35361 (H); Salo (Kisko), Haapaniemi, Plantmaannokka, calcareous rock outcrop on shore of Lake Määrjärvi, on calcareous boulder, 43 m alt., 60°12'N, 23°31'E, 4 June 2010, J. Pykälä 37746 (H); Koillismaa, Kuusamo, Oulanka National Park, Pikkukönkäänkuru, dolomite rock outcrop, on SW-facing wall, 173 m alt., 66°21'N, 29°19'E, 8 Aug 2009, J. Pykälä 35930 (H); Kuusamo, Oulanka National Park, Kiutaköngäs, N-shore of river Oulankajoki, dolomite rock outcrop, on SE-slope, 150 m alt., 66°22'N, 29°20'E, 12 Aug 2009, J. Pykälä 36308 (H); Salla, Oulanka National Park, 400 m N of Savilampi, shore of river Savinajoki, cliff, dolomite rock outcrop, on NW-facing wall, 177 m alt., 66°25'N, 29°10'E, 13 Aug 2009, J. Pykälä 36371 (H); Kuusamo, Juuma, Lammasvuoma, gorge, calciferous (dolomite) schistose rock outcrop, on NE-facing wall, 225 m alt., 66°16'N, 29°26'E, 8 Aug 2010, J. Pykälä 39475 (H), 39478 (H), 39491 (H); Salla, Oulanka National Park, Savilamminniemi, shore of lake Savilampi, cliff, dolomite rock outcrop, on E-facing wall, scarce, 185 m alt., 66°25'N, 29°10'E, 12 Aug 2010, J. Pykälä 39803 (H); Kuusamo, Oulanka National Park, Kiutaköngäs, by the rapids, shore of Oulankajoki river, calciferous (dolomite) schistose rock outcrop, N-slope, on boulder, 152 m alt., 66°22'N, 29°20'E, 18 Aug 2010, J. Pykälä 40284 (H); Kuusamo, Juuma, Oulanka National Park, Hautaniitynvuoma, gorge, dolomite rock outcrop, on high NE-facing wall, very scarce, 190 m alt., 66°15'N, 29°26'E, 21 Aug 2011, J. Pykälä 44671 (H); Kuusamo, Juuma, Oulanka National Park, Hautaniitynvuoma, gorge, stony NW-slope with sparse stunted birches, close to bottom, on dolomite boulder, 181 m alt., 66°15'N, 29°26'E, 21 Aug 2011, J. Pykälä 44734 (H); Salla, Oulanka National Park, Savilampi 1.2 km NE, steep E-slope, open area in forest, on small dolomite rock, 190 m alt., 66°26'N, 29°11'E, 23 Aug 2011, J. Pykälä 44881 (H); Pohjois-Karjala, Juuka, Polvela, Valkealampi, close by E-shore, *Pinus sylvestris*-dominated forest, calcareous rock outcrop, on W-slope, 176 m alt., 63°10'N, 29°07'E, 11 July 2011, J. Pykälä 42392 (H), 42419 (H); Juuka, Polvela, Valkealampi, close by E-shore, *Pinus sylvestris*-dominated forest, calcareous rock outcrop, W-slope, directly on rock, rather scarce, 175 m alt., 63°10'N, 29°07'E, 11 July 2011, J. Pykälä 42406 (H); Juuka, Petrovaara, Riihilahti S, shore of lake Polvijärvi, calcareous rock outcrop, on W-facing wall, 171 m alt., 63°09'N, 28°58'E, 13 July 2011, J. Pykälä 42510 (H).

#### Notes.

This species has usually been treated as *V. papillosa* Ach. and was also reported from Finland as *V. papillosa* (Pykälä 2010a). However, Orange (2004b) showed that the type specimen of *V. papillosa* belongs to *V. viridula* (Schrad.) Ach. The type specimen of *V. lacerata* is small, but it fits rather well with the Finnish material. However, ITS sequences from Central Europe are needed to confirm the identity of *V. lacerata*. According to Breuss (2008b), the exciple size in *V. lacerata* is 0.4–0.6 mm, i.e. exceeding the size of *V. subjunctiva*. The ITS phylogeny does not separate *V. subjunctiva* from *V. foveolata*. These two taxa are here kept separated pending further study (see *V. foveolata*). *Verrucaria subjunctiva* and *V. foveolata* have larger spores than the other studied species. However, there is much overlap in the spore size of *V. devergens* and *V. kuusamoensis* and specimens with suboptimally-developed spores are easily misidentified. *Verrucaria subjunctiva* has larger perithecia and longer periphysoids than *V. kuusamoensis*.

### Verrucaria subtilis

Taxon classificationFungiVerrucarialesVerrucariaceae

Müll. Arg., Flora 57: 536, 1874

C34A4EAF-7217-5331-BEAA-764F5964C4A2

= Verrucaria hypophaea (J. Steiner & Zahlbr.) Servít, Stud. Bot. Cechoslov. 11(3): 114, 1950
Verrucaria rupestris var. hypophaea J. Steiner & Zahlbr., Ann. K. K. naturh. Hofmus. Wien 22: 107, 1908.Basionym. Type. [Croatia] Hungaria; ad saxa dolomitica prope pagum Pulac supra Fiume, ca. 250 m a.s.m, leg. J. Schuler, Kryptogamie exsiccatae 1521 (M-0164001!, PRM-789449!, syntypes).
=?Verrucaria infidula Zschacke, Rabenh. Krypt.-Fl. 9(1)1: 135, 1933. Type. [Poland,] Eitner, Sammlung H. Zschacke 4708 (B-600194849!, syntype?) (see Pykälä 2016)

#### Type.

[Switzerland] Bagnes-Thal, nördl. vom Hotel Monvoisin gegen den Plaine an Dolomitfelsen 16.9.1873 (G-00295028!, syntype); … Monvoisin & Bonat Mepa in Bagnes-Thal 1874 (G-00260361!, syntype?).

#### Description.

Prothallus absent. Thallus white, grey or pale brown, endolithic, or thinly epilithic, continuous to rimose, up to 0.1 mm thick. Perithecia 0.15–0.34(–0.44) mm in diam., (1/2–)3/4(–1)-immersed, leaving shallow to deep pits in the rock, few perithecia occasionally not leaving pits, sometimes covered by a thin thalline layer except for the ostiolar region; 40–160 perithecia/cm^2^. Ostiole inconspicuous, tiny, pale or dark, plane or depressed, in two specimens, several ostioles slightly projecting, ca. 20–40(–70) mm wide. Involucrellum apical or covering half of the exciple, rarely in few perithecia exceeding half of the exciple, 30–70(–80) mm thick, appressed to the exciple to clearly diverging from the exciple. Exciple 0.16–0.33 mm in diam., wall pale or pale brown (rather rare), usually dark brown or black, 18–30 mm thick. Periphysoids ca. 20–40(–50) × (1–)1.5–2.5(–3) mm, branching. Asci 58–84 × 22–28 mm, 8-spored. Ascospores 0-septate, (19.8–)22.9–25.2–27.4(–30.7) × (8.3–)9.6–10.5–11.4(–12.8) mm (n = 400), perispore 1 mm thick.

#### Habitat and distribution.

The species grows on various calcareous rocks and in lime quarries. It occurs both in sun-exposed and shady habitats. It is amongst the most common species of *Verrucaria* on calcareous rocks of southern Finland. It may occur in the whole country, but the northernmost sequenced specimens are from the biogeographical province of Koillismaa. In Finland, *V. subtilis* is the most common species of *Verrucaria* belonging to the *Thelidium* group and having perithecia leaving pits in the rock.

#### Other specimens examined.

Finland. Varsinais-Suomi, Lohja, Paavola, N of Rautaniemi, stony SE-slope, young *Pinus sylvestris*-plantation, on calcareous stone, 50 m alt., 60°13'N, 23°54'E, 21 May 2005, J. Pykälä 26865 (H); Pohja, Kuovila, 150 m NW of Valkjärvi, small rather flat calcareous rock outcrop, 50 m alt., 60°08'N, 23°23'E, 9 October 2006, J. Pykälä 29589 (H); Karkkila, Haavisto, 200 m N of Saaressuo, on calcareous rock outcrop, 132 m alt., 60°31'N, 24°22'E, 24 May 2008, J. Pykälä 32606 (H); Suomusjärvi, Sallittu, Huuttavanmäki, S-slope, on calciferous boulder, 110 m alt., 60°18'N, 23°37'E, 28 June 2008, J. Pykälä 32749 (H); Salo (Kiikala), Saari, Kalkkimäki, abandoned lime quarry, on NW-facing wall, 105 m alt., 60°25'N, 23°40'E, 4 July 2009, J. Pykälä 34601 (H); Salo (Kisko), Haapaniemi, Multsilta, calcareous rock outcrop, on shady N-facing wall, 65 m alt., 60°13'N, 23°29'E, 17 July 2009, J. Pykälä 35093 (H); Kemiönsaari (Västanfjärd), Billböle, Svinberget, calcareous rock outcrop, on W-slope, st pc, 25 m alt., 60°03'N, 22°43'E, 4 Sept 2009, J. Pykälä 36819 (H); Länsi-Turunmaa (Parainen), Hyvilemp, Hyvilemp, abandoned lime quarry, on SW-facing wall, scarce, 15 m alt., 60°17'N, 22°12'E, 14 Sept 2009, J. Pykälä 37102 (H); Karjalohja, Pyöli, E of Innoonlampi, rocky forest, on calcareous boulder, 46 m alt., 60°13'N, 23°49'E, 28 Sept 2009, J. Pykälä 37329 (H), 37331 (H); Salo (Kisko), Haapaniemi, Sorronniemi, abandoned lime quarry, on SE-facing wall, scarce, 65 m alt., 60°13'N, 23°30'E, 4 June 2010, J. Pykälä 37794 (H);

Salo (Kisko), Jyly, 200 m NE of Purslammi, calcareous rock outcrop, on NW-facing wall, 68 m alt., 60°14'N, 23°36'E, 17 June 2010, J. Pykälä 38140 (H); Salo (Särkisalo), Kaukosalo, Pyölinmäki, abandoned lime quarry, quarry spoil heap, NW-slope, on calcareous pebbles, 15 m alt., 60°07'N, 22°58'E, 17 June 2011, J. Pykälä 42225 (H); Koillismaa, Salla, Oulanka National Park, Pikkuköngäs, shore of river Oulankajoki, high cliff, dolomite rock outcrop, on SW-slope, 180 m alt., 66°25'N, 29°08'E, 13 Aug 2010, J. Pykälä 39870 (H); Kuusamo, Liikasenvaara, Iso Sirkkalampi 200 m E, SW-slope, young *Larix*-plantation, on dolomite boulder, rather scarce, 295 m alt., 66°21'N, 29°35'E, 18 Aug 2010, J. Pykälä 40280 (H); Salla, Oulanka National Park, Savilampi 850 m N, shore of Savinajoki river, river shore, on dolomite boulder, on S-facing wall, 182 m alt., 66°26'N, 29°10'E, 23 Aug 2011, J. Pykälä 44843 (H), 44844 (H); Keski-Pohjanmaa, Vimpeli, Vimpeli, Ryytimaa, lime quarry, quarry spoil heap, young deciduous forest, on calcareous boulders, rather scarce, 135 m alt., 63°09'N, 24°01'E, 31 Aug 2010, J. Pykälä 40596 (H);

Vimpeli, Vimpeli, Ryytimaa, lime quarry, S-slope, on pebbles, 125 m alt., 63°09'N, 24°01'E, 2 Sept 2010, J. Pykälä 40833 (H); Vimpeli, Möksy, Kotakangas, abandoned lime quarry, small quarry spoil heap, on pebbles, 122 m alt., 63°07'N, 23°58'E, 2 Sept 2010, J. Pykälä 40859 (H); Vimpeli, Möksy, Kotakangas, by abandoned lime quarry, quarry spoil heap, W-slope, on boulders, 120 m alt., 63°07'N, 23°58'E, 2 Sept 2010, J. Pykälä 40874 (H); Uusimaa, Vantaa, Sotunki, Bisa, 300 m E-NE, herb-rich forest, abandoned lime quarry, on SW-facing wall, 35 m alt., 60°17'N, 25°09'E, 7 June 2011, J. Pykälä 41857 (H); Pohjois-Karjala, Juuka, Nunnanlahti, Mustanvaara, dolomite rock outcrop, on SE-slope, 140 m alt., 63°09'N, 29°09'E, 14 July 2011, J. Pykälä 42540 (H); Etelä-Savo, Kerimäki, Ruokojärvi, Pitkäniemi, abandoned lime quarry, gravelly field, on calcareous pebbles, 85 m alt., 61°56'N, 29°00'E, 15 Sept 2011, J. Pykälä 45794 (H), 45817 (H), 45847 (H).

#### Notes.

*Verrucaria subtilis* may be confused with several other species treated in this paper (see descriptions of these species). *Verrucaria cavernarum* and *V. difficilis* may differ by often longer involucrellum and slightly larger spores. The species may also be mixed up with *Verrucaria epilithea* Vain. and *Verrucaria muralis* Ach. These species have shorter spores (17–26 mm long) and the perithecia are not leaving pits in the rock or the pits are shallow. The first specimens of *V. subtilis* from Finland were identified as *Verrucaria mimicrans* Servít and *V. transfugiens* Zschacke (Pykälä and Breuss 2008). The type material of *V. mimicrans* has not been located and the identity of this species remains to be studied. *Verrucaria transfugiens* (see Pykälä 2016) differs in smaller spores and the presence of dark lines between contiguous conspecific thalli. *Verrucaria hypophaea* has usually been considered to belong to *V. muralis* or to *V. schindleri* Servít, which is said to differ from *V. muralis* by a dark exciple (Breuss 2007). However, *V. hypophaea* clearly differs from *V. muralis* by larger spores and the perithecia commonly leaving deep pits in the rock. The characters of *V. hypophaea* fit well with *V. subtilis*.

### Verrucaria vacillans

Taxon classificationFungiVerrucarialesVerrucariaceae

Pykälä & Myllys sp. nov.

FAEF26ED-6270-581B-8803-B6DAFF75F133

835675

Fig. 2G

#### Diagnosis.

Species characterised by dark lines between contiguous conspecific thalli, pale usually endolithic thallus, perithecia leaving shallow to deep pits in the rock, very variable involucrellum, ascospores (18–)23–28(–32) × (8–)11–13(–15) mm, morphologically rather similar to the Finnish species of the *V. subtilis* complex, but the sequence divergence in ITS 4.5–6.8%.

#### Holotype.

Finland. Enontekiön Lappi, Enontekiö, Porojärvet, Toskalharji, Toskalpahta, fell, SW-slope, scree, on dolomite boulder, 795 m alt., 69°11'N, 21°29'E, 1 Aug 2011, J. Pykälä 43118 (H9205851, GenBank accession number: MT229829).

#### Description.

Prothallus absent. Thallus white, whitish grey or pale brownish, mainly endolithic to thinly epilithic, 20–170 mm thick, algal cells 5–10 mm, contiguous conspecific thalli separated by dark lines, 0.21–0.41 mm wide. Perithecia 0.15–0.47 mm in diam., 1/4–3/4-immersed, usually leaving shallow to fairly deep pits in the rock, rarely few perithecia not leaving pits, often surrounded by a thalline collar, 60–160(–200) perithecia/cm^2^. Ostiole tiny or conspicuous, pale to dark, plane or depressed, ca. 20–40(–60) mm wide, wider ostiolar depression occasionally present, up to 160 mm wide. Involucrellum apical, covering half of the exciple, exceeding half of the exciple or rarely to the exciple base, 30–70(–90) mm thick, appressed to the exciple, moderately diverging from the exciple, strongly diverging from the exciple or even spreading outwards away from the exciple. Exciple 0.15–0.26 mm, wall dark brown or black, 17–35 mm thick. Periphysoids ca. 25–40(–50) × 1.5–2.5 mm, branching. Asci 67–84 × 27–28 mm, 8-spored. Ascospores 0-septate, (18.1–)22.7–25.3–28.0(–31.7) × (8.3–)10.8–11.9–13.1(–15.2) mm (n = 228), perispore 1–1.5 mm thick.

#### Habitat and distribution.

The species is restricted in Finland to the calcareous mountains (Scandes) in NW Finland above the tree level. It always grows on dolomite. It grows on rock outcrops, boulders, stones and pebbles.

#### Etymology.

The specific epithet refers to the high morphological variation in the involucrellum from apical to (rarely) reaching the exciple base level, from being appressed to the exciple to spreading outwards away from the exciple and from fairly thin to thick.

#### Other specimens examined.

Finland. Enontekiön Lappi, Enontekiö, Porojärvet, Toskalharji, Toskalpahta, fell, SW-slope, scree, on dolomite pebbles, 785 m alt., 69°11'N, 21°29'E, 1 Aug 2011, J. Pykälä 43058 (H); Enontekiö, Porojärvet, Toskalharji, Toskaljärvi N, fell, gentle SE-slope, dolomite rock outcrop, on dolomite stones, with *V. foveolata*, 730 m alt., 69°12'N, 21°26'E, 2 Aug 2011, J. Pykälä 43232 (H); Enontekiö, Porojärvet, Toskalharji, Toskaljärvi N, fell, dolomite rock, gentle S-slope, on dolomite stone, 720 m alt., 69°12'N, 21°26'E, 2 Aug 2011, J. Pykälä 43272 (H); Enontekiö, Porojärvet, Toskalharji, Toskaljärvi N, fell, dolomite rock, on SE-facing wall, 720 m alt., 69°12'N, 21°26'E, 2 Aug 2011, J. Pykälä 43296 (H); Enontekiö, Porojärvet, Toskalharji, Toskaljärvi N, fell, dolomite rock, gentle SE-slope, on dolomite pebbles, 730 m alt., 69°12'N, 21°26'E, 2 Aug 2011, J. Pykälä 43302 (H); Enontekiö, Porojärvet, Toskalharji, Toskaljärvi N, fell, dolomite scree, on dolomite boulder, rather abundant, 710 m alt., 69°11'N, 21°26'E, 2 Aug 2011, J. Pykälä 43384 (H); Enontekiö, Kilpisjärvi, Saana, nature reserve, E-part, fell, dolomite rock, on SW-facing wall, 880 m alt., 69°02'N, 20°51'E, 10 Aug 2011, J. Pykälä 44075, 44081b (H); Enontekiö, Kilpisjärvi, Saana, fell, steep NE-slope, dolomite rock, on NE-facing wall, 820 m alt., 69°02'N, 20°51'E, 11 Aug 2011, J. Pykälä 44142, 44162 (H); Enontekiö, Kilpisjärvi, Saana, nature reserve, E-part, fell, steep SW-slope, dolomite rock, on SW-facing wall, 730 m alt., 69°02'N, 20°51'E, 12 Aug 2011, J. Pykälä 44255 (H).

#### Notes.

Based on ITS sequences, *V. vacillans* is genetically well distinct from other *Verrucaria* species. However, it may be confused with several other species. *Verrucaria vacillans* is most difficult to separate from *V. cavernarum*, *V. difficilis* and *V. subtilis*. In these three species, dark lines between contiguous conspecific thalli are never present. *Verrucaria cavernarum* and *V. subtilis* have an involucrellum seldom exceeding half of the exciple (and then only in a minority of perithecia). The exciple of *V. subtilis* is sometimes pale (although usually dark). The spores tend to be slightly broader in *V. vacillans* than in *V. subtilis*. However, specimens of *V. vacillans* without dark lines and with a short involucrellum may not be possible to separate from *V. cavernarum* and *V. subtilis* by morphology. Specimens of *V. vacillans* with a deep reaching involucrellum may not be separable from *V. difficilis* if dark lines are absent. *Verrucaria vacillans* may also be confused with *V. devergens*, *V. kuusamoensis*, *V. epilithea* and *V. muralis*. *Verrucaria kuusamoensis* has an involucrellum usually exceeding half of the exciple, larger spores and dark lines are rather rare. *Verrucaria devergens* has larger spores and the involucrellum is usually absent or sometimes apical. *Verrucaria epilithea* and *V. muralis* have perithecia not leaving pits or the pits are shallow, the spores do not exceed 26 mm in length and dark lines are absent.

##### Names considered inapplicable to the species treated above

### Verrucaria adelminienii

Taxon classificationFungiVerrucarialesVerrucariaceae

Zschacke, Rabenh. Krypt.-Fl. 9(1) 1: 160, 1933

817EEAC3-F51C-5662-B73F-FC3A3EE0D1AC

#### Type.

France, Cantal: Auf hartem Kalk bei St. Santin, 1886, F. Adelminien (B600191351!, syntype).

#### Notes.

The specimen in B is tiny with ca. 10 perithecia, of which all but two are covered by glue. The specimen is not identifiable and the species is better to be treated as a species with unresolved status (Pykälä 2016).

### Verrucaria aljazevi

Taxon classificationFungiVerrucarialesVerrucariaceae

Servít, Stud. Bot. Čech. 9: 71, 1948

346790E0-EE99-5FD6-845F-73308FCDA09A

#### Type.

[Slovenia] Carniola, Mojakrana, Aljazev dom, 1100 m, 1931, Servít (PRM-858477!, holotype?).

#### Description.

Prothallus not seen. Thallus white, endolithic. Perithecia 0.11–0.36 mm, immersed, leaving deep pits in the rock. Involucrellum absent. Exciple ca. 0.25 mm in diam., wall dark. Periphysoids ca. 25–35 × 2–3 mm, sparsely branching, *Bagliettoa*-like. Ascospores 0-septate (only few seen), 15–18 × 6–8 mm.

#### Notes.

According to the protologue (Servít 1948), the spore size is 20–28 × 7–8 mm. The species may be related to *Bagliettoa calciseda* (DC.) Gueidan & Cl. Roux.

### Verrucaria alpigena

Taxon classificationFungiVerrucarialesVerrucariaceae

Breuss, nom. nov., Sauteria 15: 122, 2008

4ECAE20A-B460-580B-98DD-B07C9632F072

#### Type.

Austria, Niederösterreich, Voralpen, Bez. Lilienfeld, Gem. Kleinzell, SE von Salzerbad, Weg von Reintal zum Kruckensattel, 550–650 m alt., 29.3.2002, O. Breuss (8060) 19.990 (LI-01763881!, holotype).

#### Description.

Prothallus rather weakly developed, medium brown, weakly fimbriate. Thallus pale greyish-brown with frequent medium brown flecks, rimose, ca. 0.05–0.15 mm thick. Perithecia 0.22–0.38 mm, 1/2–3/4-immersed, not leaving pits to leaving shallow pits in the rock, thinly thalline covered except apex; ca. 80–100 perithecia cm^2^. Ostiole pale brown, plane, ca. 20–60 mm wide. Involucrellum to the exciple base level, occasionally enveloping the exciple, ca. 40–60 mm thick, appressed to the exciple. Exciple 0.21–0.24 mm in diam., wall pale to dark brown. Ascospores 0-septate, (22.7–)26.1–28.1– 30.9(–33.6) × (12.1–)12.4–13.5–14.5(–15.8) mm (n = 20).

#### Notes.

This species was erroneously reported from Finland by Pykälä (2011), but based on the ITS phylogeny, the specimen belongs to *V. subjunctiva*. It differs from the other Finnish specimens of *V. subjunctiva* by the pale exciple wall. Originally, *V. alpigena* was described as a species related to *V. muralis*, but differing by larger spores (Breuss 2008). Studying the type specimen of *V. alpigena* revealed that the species may not be related to *V. muralis* nor to the *Thelidium* group. It has a superficial morphological similarity to *Verrucaria ahtii* Pykälä, Launis & Myllys (Pykälä et al. 2017), but the spores are larger. *Verrucaria alpigena* may belong to the so-called Endocarpon group such as *V. ahtii*.

### Verrucaria bavarica

Taxon classificationFungiVerrucarialesVerrucariaceae

Servít, Stud. Bot. Čech. 9: 73, 1948

22273CBD-DD48-5F7A-94A7-67B44D31A7C3

#### Type.

[Germany,] Alg. Alpen, Britzelmeyer (PRM-858488!, holotype?).

#### Description.

Prothallus not seen. Thallus whitish grey, endolithic. Perithecia 0.22–0.36 mm, 3/4–1-immersed, leaving deep pits in the rock. Involucrellum apical, ca. 60 mm thick, appressed to the exciple. Exciple ca. 0.24 mm in diam., wall dark. Ascospores 0-septate, 23–31 × 11–13 mm.

#### Notes.

The specimen is small and only one perithecium was dissected. Our spore measurements match well with the original description (26–32 × 10–12(–14) mm, according to Servít 1948), as well as the values given by Breuss (26–32 × 10–13 mm, according to Breuss 2016). According to Servít (1948), the size of the exciple is 0.4 mm. *Verrucaria bavarica* is morphologically close to *V. cavernarum* and *V. subtilis*, but may differ in having a larger exciple (which was not confirmed due to the very small size of the specimen) and slightly larger spores.

### Verrucaria caesiopsila

Taxon classificationFungiVerrucarialesVerrucariaceae

Anzi, Comm. Soc. Critt. Ital. 2(1): 23, 1864

B13A8EC9-9A5E-5830-B605-9B0F1419B1FE

#### Type.

[Switzerland] ad saxa dolomitica in alpe Camsciano sopra Poschiavo, Anzi nro. 364 (S-L140!, syntype).

#### Description.

Prothallus not seen. Thallus inconspicuous, endolithic. Perithecia 0.15–0.25 mm, 3/4–1-immersed, leaving deep pits in the rock. Involucrellum absent. Exciple ca. 0.25–0.3 mm in diam., wall black. Periphysoids ca. 30–40 × 2 mm. Ascospores 0-septate, 17–23 × 11–12(–14) mm.

#### Notes.

The species differs from *V. devergens*, *V. foveolata* and other species treated in the Taxonomy section in smaller spores.

### Verrucaria carnea

Taxon classificationFungiVerrucarialesVerrucariaceae

(Arnold) Servít, Stud. Bot. Čechoslov. 9: 77, 1948

A746ADF9-B058-5585-BBC6-FD8D50100CD8

Verrucaria leightonii var. carnea Arnold in Zwackh, Flora 47: 87, 1864. Basionym.

#### Type.

[Germany] an einer Sandsteinmauer in den Weinbergen bei Neuenheim, Febr. 1863, W. von Zwackh (M-0023494!, syntype?).

#### Description.

Prothallus not seen. Thallus pale grey, rimose to areolate, areoles 0.3–0.7 mm. Perithecia 0.22–0.26 mm, immersed in thallus. Involucrellum absent. Exciple wall pale. Periphysoids ca. 50–80 × 2.5–3 mm, branching. Ascospores 0-septate (only few seen), 20–28 × 13–14 mm.

#### Notes.

Krzewicka (2012) treated *V. carnea* as a pigment-deficient mutant of *V. hochstetteri* and Gilbert (1996) as an albino form of *V. macrostoma* Dufour ex DC. However, it differs in several morphological characters from these species. Oïhénart et al. (2018) accepted *V. carnea* as a distinct species.

### Verrucaria cinereorufa

Taxon classificationFungiVerrucarialesVerrucariaceae

Schaer., Lich. Helv. Spicil. 7, 338, 1836

84023B54-8011-569C-A41F-F02B8D214948

#### Type.

[France,] Saléve (H-NYL3038!, UPS!, probably syntypes).

#### Description.

Prothallus not seen. Thallus pale greyish-brown, thinly epilithic, continuous. Perithecia 0.38–0.61 mm, 1/2–3/4-immersed, leaving fairly deep to deep pits in the rock; ca. 30–60 perithecia/cm^2^. Ostiole plane to depressed, ca. 20–60 mm wide. Involucrellum covering half of the exciple, ca. 70–180 mm thick. Exciple 0.23–0.38 mm in diam., wall black. Periphysoids long, ca. 1.5–3 mm thick. Ascospores 0-septate, 30–38 × 12–15 mm.

#### Notes.

The species may be related to *Verrucaria depressula* Servít, but has larger perithecia and thicker involucrellum. The species was erroneously reported by Pykälä (2010a) from Finland.

### Verrucaria clauzadei

Taxon classificationFungiVerrucarialesVerrucariaceae

de Lesd., Bull. Bot. Soc. France 97: 171, 1950

CC22F1BB-C8F7-56B8-9C97-1050CFD7E52B

#### Type.

[France] Calcaire argileux enposé au N, á 100 m au NE du pas du Bourreau Allaunch, 7.7.1951, Clauzade (PRM-858628!, syntype?).

#### Description.

Prothallus not seen (but, according to the protologue, “linea nigra marginatus”). Thallus grey with tiny brown flecks, thinly epilithic, continuous. Perithecia 0.25–0.45 mm, 3/4–1-immersed, leaving deep pits in the rock; ca. 70–80 perithecia/cm^2^. Involucrellum covering half of the exciple, ca. 60–80 mm thick. Exciple ca. 0.25 mm in diam., wall black. Periphysoids ca. 35–50 × 2–2.5 mm. Ascospores 0-septate, 28–34(–38) × 12–13 mm.

#### Notes.

The studied specimen is tiny and better material is needed to solve the identity of the species. The specimen matches in most respects with *V. subjunctiva*. The spores seen were narrower, but the spore size given in the protologue (Bouly de Lesdain 1950) 25–33 × 13–16 mm is similar to *V. subjunctiva*.

### Verrucaria cryptica

Taxon classificationFungiVerrucarialesVerrucariaceae

(Arnold) J. Steiner, nom. inval., Verh. zool.-bot. Ges. Wien 61: 41, 1911

78AD11B7-5FFB-5A19-8A61-38B47AC9BC7B

Amphoridium crypticum Arnold, nom. inval., Lich. Frank. Jura 257, 1885. Basionym.

#### Type.

[Italy] An Kalksteinen einer Schutthalde unterhalb der Kalkwände an der Südseite des Latemar –Gebirges oberhalb Predazzo, Südtirol, 21. Aug. 1883, Arnold (H-NYL 7009!, H!, UPS-L-169663!, isotypes).

#### Description.

Prothallus absent. Thallus endolithic, grey. Perithecia 0.15–0.39 mm, (3/4–)1-immersed in rock, leaving deep pits in the rock. Involucrellum absent or possibly in some perithecia, small apical involucrellum. Exciple ca. 0.25–0.40 mm in diam., apex thickened, wall black, ca. 30 mm thick. Periphysoids ca. 50–70 × 2 mm, branched-anastomosing. Ascospores 0-septate, 25–30(–32) × (12–)13–16(–17) mm, perispore 1(–1.5) mm thick, persistent.

#### Notes.

The species is rather similar to *V. foveolata*, but the halonate perispore seems to be persistent. This species seems not to have been validly published as the species description is missing from Arnold (1885) and Steiner (1911).

### Verrucaria depressula

Taxon classificationFungiVerrucarialesVerrucariaceae

Servít, nom. nov., Stud. Bot. Čechoslov. 9: 80, 1948

83DD907A-0E16-54B6-9A19-010049FC54D5

= Verrucaria depressa Stenhammar, nom. illeg. non Meyen & Flot., Öfvers. K. Svensk. Vetensk.-Akad. Förhandl. 14: 120, 1857. Type. Sweden, Gotland, Lojsta, Lojsta, in collybus calcareis, 1846–55, C. Stenhammar (H!, two syntypes)
= Verrucaria obscura Th. Fr., nom. illeg. non (Sm. & Sowerby) Borrer 1836, Öfvers. K. Svensk. Vetensk.-Akad. Förhandl. 21: 276, 1865. Type. Sweden, Resmo, C. Stenhammar (UPS!, syntype)

#### Description.

Prothallus not seen. Thallus grey, pale brown, medium brown or rarely dark brown, with a violet tinge, continuous, rimose or areolate, thallus colour may be variable within specimen, 0.05–0.2(–0.3) mm thick, contiguous conspecific thalli separated by dark lines. Perithecia 0.26–0.52 mm, (1/2–)3/4-immersed, leaving shallow to deep pits in the rock; ca. 60–120 perithecia/cm^2^. Involucrellum apical, strongly diverging from the exciple (mainly spreading outwards away from the exciple), (40–) 50–90 μm thick. Exciple 0.25–0.4 mm in diameter, pale or dark. Periphysoids ca. 40–60 × (1–)1.5–2 μm. Ascospores 0-septate, (24–)27–35(–45) × 10–18(–20) μm, few spores 1-septate.

#### Notes.

Based on morphology, *V. depressula* may belong to the *Thelidium* group or perhaps more probably be related to *V. viridula*. The type locality is in Sweden and rather close to Finland, but nevertheless, no specimens fitting with *V. depressula* have been found from Finland.

### Verrucaria dermatoidea

Taxon classificationFungiVerrucarialesVerrucariaceae

(A. Massal.) Servít, Stud. Bot. Čechoslov. 9: 81, 1948

13341AE5-72AD-53CC-A285-8056945070EB

Verrucaria veronensis f. dermatoidea A. Massal., Anzi, Lich. exs. minus rari Italiae superioris 377. Basionym.

#### Type.

[Italy] ad saxa calcarea prope Veronam Mass., Anzi, Lich. Exs. minus rari Italiae superioris 377 (UPS!, syntype).

#### Description.

Prothallus not seen. Thallus grey, rimose to areolate, 0.2–0.4 mm thick. Perithecia 0.18–0.23 mm, immersed in thallus. Involucrellum apical, ca. 30–40 mm thick. Exciple ca. 0.4–0.45 mm in diam., pear-shaped, wall black. Ascospores 0-septate, 27–32 × 13–15 mm.

#### Notes.

The studied specimen is conspecific with *V. viridula*.

### Verrucaria dolomitica

Taxon classificationFungiVerrucarialesVerrucariaceae

(A. Massal.) Kremp., Denkschrft. K. Bayer. Bot. Gesellsch. 4(2): 238, 1861

DDBB22B2-D610-50E7-B740-3CB30B6DEBF4

Amphoridium dolomiticum A. Massal., Symmict. Lich. 80, 1855. Basionym.

#### Type.

[Italy,] in op. Giazza ad saxa dolomitica, 1853, A. Massalongo (VER!, syntype); Ad saxa dolomitica in oppido Giazza Prov. Veron. Massal., Massalongo Lichens Ital. Exsiccatae 250 (VER!, syntype).

#### Description.

Prothallus not seen. Thallus pale greyish-cream, endolithic to thinly epilithic surrounding perithecia, slightly rimose, thalli bordered by a blackish-brown line. Perithecia 0.26–0.53 mm, (1/2–)3/4-immersed, leaving deep pits in the rock; 70–100 perithecia/cm^2^. Ostiole, pale, plane or depressed, ca. 40–150 mm wide. Involucrellum apical, 50–80 mm thick. Exciple 0.24–0.42 mm in diam., wall medium brown to blackish-brown, pale in one studied perithecium. Periphysoids ca. 40–50 × 2 mm. Ascospores 0-septate, 26–37 × 11–18 mm.

#### Notes.

*Verrucaria dolomitica* and *V. foveolata* have been treated as separate taxa because of the presence (in the former) or absence (in the latter) of an apical involucrellum (Breuss 2004). *Verrucaria dolomitica* was reported as new to Finland by Pykälä (2010b). However, the Finnish specimens with and without an apical involucrellum have identical ITS sequences or the sequences differ only by a few bases. The Finnish specimens originally identified as *V. dolomitica* are conspecific with *V. foveolata* and treated as such in Stenroos et al. (2016). The Finnish specimens identified as *V. dolomitica* usually have endolithic thalli and no dark lines have been observed between thalli. The syntypes of *V. dolomitica* studied have a partly epilithic thin thallus bordered by a dark line. Furthermore, the perithecia are larger than in the Finnish specimens. Thus, *V. dolomitica* is apparently distinct from *V. foveolata*, but *V. dolomitica* does not occur in Finland.

### Verrucaria epipolaea

Taxon classificationFungiVerrucarialesVerrucariaceae

Ach., Lichenogr. Univers. p. 285, 1810

32D3583F-B432-5259-B788-5C74E6D2E51C

#### Type.

[Switzerland] Helvetia (H-ACH 686!, holotype?, piece on the upper left).

#### Description.

Prothallus not seen. Thallus pale grey, thin, continuous, up to 0.1 mm thick. Perithecia 0.26–0.41 mm, 1/4–1/2-immersed in thallus, sometimes with thin irregular thalline cover; ca. 70–100 perithecia/cm^2^. Ostiole inconspicuous, dark, plane or depressed, ca. 20–70 mm wide. Involucrellum slightly exceeding half of the exciple or reaching the exciple base level, 50–70 mm thick, appressed to the exciple or slightly diverging from it. Exciple 0.25–0.32 mm in diam., wall pale. Periphysoids ca. 30–40 × 1.5–2 mm, branching. Asci 75–117 × 25–37 mm, 8-spored. Ascospores 0-septate, (25.3–)26.8–29.6–32.3(–34.4) × (10.4–)11.8–12.5–13.2(–13.4) mm (n = 37).

#### Notes.

*Verrucaria epipolaea* is reminiscent of rare morphs of *V. kuusamoensis* with a pale exciple. It differs in less immersed perithecia by not leaving pits and in the consistently hyaline exciple wall.

### Verrucaria euboensis

Taxon classificationFungiVerrucarialesVerrucariaceae

Servít, Stud. Bot. Čech. 9: 83, 1948

01B55C54-94DE-52C3-A163-11ADF2BB32D4

#### Type.

[Greece,] Euboea: Berg Xerowuni, ca. 1100 m alt., 1931, Rechinger (PRM-858655!, isotype).

#### Description.

Prothallus not seen. Thallus white to whitish-grey, endolithic. Perithecia 0.2–0.35 mm, 3/4–1-immersed, leaving deep pits in the rock. Involucrellum covering half of the exciple, ca. 100 mm thick, appressed to the exciple. Exciple ca. 0.35–0.45 mm in diam., wall black. Periphysoids ca. 40–50 × 2–2.5 mm. Ascospores 0-septate, 25–30 × 12–16 mm.

#### Notes.

The species may be conspecific with *V. viridula*, but the thallus is endolithic.

### Verrucaria grossa

Taxon classificationFungiVerrucarialesVerrucariaceae

Nyl., in Nylander & Saelan, Herb. Mus. Fennici 111, 1859

7689CA17-CA34-59E8-9B82-E17DED837634

#### Type.

[Russia,] Lapponia Rossica, 1843, F. Nylander (H!, holotype or syntype).

#### Description.

Prothallus not seen. Thallus grey, rimose. Perithecia ca. 0.4–0.6 mm, 1/2-immersed, leaving deep pits in the rock. Involucrellum enveloping the exciple, ca. 50–100 mm thick, thicker at apex. Exciple ca. 0.3–0.4 mm in diam., wall black. Ascospores in very poor condition, 0-septate, ca. 22–25 × 10 mm.

#### Notes.

The specimen in H is small and in a very poor condition. Vainio (1921) reported the spore size of 15–24 × 10–18 mm. Pykälä (2010b) reported the species new to Finland. The Finnish specimen (the sequencing failed) may be rather similar to the one sectioned perithecium of the type of *V. grossa*, which, however, had spores in poor condition. *Verrucaria grossa* should be treated as a species with unresolved identity unless better type material can be located.

### Verrucaria hercegensis

Taxon classificationFungiVerrucarialesVerrucariaceae

Servít, Stud. Bot. Čech. 9: 85, 1948

8D6A1538-ADEA-574A-BB03-D59DEF27C6B5

#### Type.

[Montenegro] Dalmatia mer., Herceg Novi, 80 m, 1929, M. Servít (PRM-760604!, holotype).

#### Description.

Prothallus not seen. Thallus white to grey, endolithic. Perithecia 0.12–0.26 mm, immersed, leaving deep pits in the rock; ca. 30–40 perithecia/cm^2^. Involucrellum absent. Exciple ca. 0.4 mm in diam., wall black. Periphysoids ca. 35–50 × 1.5–2 mm, branched-anastomosing. Ascospores 0-septate (only few seen), 20–23 × 10–11 mm.

#### Notes.

The spore size given in the protologue (Servít 1948) is 21–25(–32) × 12–13(–15) mm. The species differs from *V. devergens* and *V. foveolata* in smaller spores. *Verrucaria devergens* has smaller exciple (up to 0.35 mm). *Verrucaria caesiopsila* may differ in a smaller exciple and possibly in smaller spores.

### Verrucaria hochstetteri

Taxon classificationFungiVerrucarialesVerrucariaceae

Fr., Lichenogr. Europ. Reform. 435, 1831

E0BC266A-797E-5068-838F-F33A01785042

#### Type.

Germania: Regni Würtemberg, Blabyrae, ad rupes calcareas, Hochstetter (UPS-L-708716!, holotype).

#### Description.

Prothallus not seen. Thallus light grey, endolithic, dark lines between contiguous conspecific thalli present. Perithecia immersed, leaving deep pits in the rock. Involucrellum absent. Exciple 0.32–0.4 mm in diam., longer than wide, wall black, rather thin. Asci ca. 110–125 × 30–38 mm. Ascospores 0-septate, (25–)26–30(–35) × 16–20 mm.

#### Notes.

The specimen is small and the description above is based on only one perithecium dissected. *Verrucaria hochstetteri* was reported from Finland by Pykälä (2007). However, all the Finnish specimens have narrower spores (max. 18 mm broad) than in the type specimen of *V. hochstetteri* and no dark lines between contiguous conspecific thalli. Thus, they apparently do not belong to *V. hochstetteri*, but to *V. foveolata*.

### Verrucaria integra

Taxon classificationFungiVerrucarialesVerrucariaceae

(Nyl.) Nyl., Notiser ur Sällskapets pro Fauna et Flora Fennica Förhandlingar, 5: 276, 1861

09FA7969-B41C-5E27-8A5B-2E73CF1DB0BD

Verrucaria rupestris var. integra Nyl., Actes Soc. Linn. Bordeaux, 21: 183, 1856. Basionym.

#### Type.

Not in H-NYL, protologue: “in Gallia passim (Ejus statum ochraceo-tinctum, E Cebennis inferioribus in hb. Mougeot vidi)”.

#### Notes.

The type material has not been located (possibly in Paris). Nylander had a very wide circumscription for *V. integra*. Specimens identified by Nylander as *V. integra* in H-NYL represent several species of *Verrucaria*. Thus, the identity of *V. integra* cannot be solved without studying the type material. Two old records of this species have been reported from Finland (Vainio 1921), but these specimens belong to *V. viridula*. Based on Vainio’s interpretation of *V. integra*, the species may be conspecific with *V. viridula*.

### Verrucaria integrella

Taxon classificationFungiVerrucarialesVerrucariaceae

(Nyl.) Nyl., Lich. Pyrenaeorum Orient. Obs. Nov.: 21, 1891

F978A223-332E-54AB-B540-FB8908240935

Verrucaria integra (Nyl.) Nyl. f. integrella Nyl, Flora 64: 457, 1881. Basionym.

#### Type.

[Switzerland] ad dolomit supra Poschiavo, Anzi (H-NYL 3384!, syntype).

#### Description.

Prothallus absent. Thallus inconspicuous, endolithic. Perithecia 0.18–0.23 mm, 3/4–1-immersed, leaving deep pits in the rock; ca. 100–110 perithecia/cm^2^. Ostiole depressed, ca. 20–50 mm wide. Involucrellum absent (?). Exciple ca. 0.2 mm in diam., wall dark. Ascospores 0-septate, ca. 17–21 × 11–12 mm.

#### Notes.

The studied specimen may be a tiny syntype. Nylander has annotated to the specimen: spores 18–24 × 11–14 mm. *Verrucaria integrella* may be synonymous with *V. caesiopsila* as often stated in literature (e.g. Clauzade and Roux 1985; Santesson et al. 2004).

### Verrucaria koerberi

Taxon classificationFungiVerrucarialesVerrucariaceae

Hepp, Flechten Eur. 692, 1860

A350DDF5-3AED-506F-8B28-62C2B66F4E3C

#### Type.

[Germany] an Dolomitfelsen in Laubwäldern bei Eichstätt (Baiern), F. Arnold, Hepp, Flechten Eur. 692 (UPS-L-069713!, syntype).

#### Description.

Prothallus not seen. Thallus white, endolithic to thinly epilithic. Perithecia 0.2–0.3 mm, 3/4–1-immersed, leaving deep pits in the rock, surrounded by a thalline collar, ca. 50–120 perithecia/cm^2^. Ostiole inconspicuous, dark, depressed, ostiolar depression up to 100 mm wide. Involucrellum apical, 40–70 mm thick, appressed to the exciple. Exciple 0.17–0.25 mm in diam., wall dark. Periphysoids ca. 30 × 1.5 mm. Ascospores 0-septate, (17–)18–21 × (7–)8 mm.

#### Notes.

This species differs from *V. subtilis* in smaller spores. The specimen H-NYL 7012 does not belong to the type material because it has too large spores (25–33 × 12–16 mm).

### Verrucaria mastoidea

Taxon classificationFungiVerrucarialesVerrucariaceae

(A. Massal.) Trevis., Conspect. Verruc. p. 8, 1860

91F4B156-56CA-5AFC-ADBC-52B7BA34B06C

Amphoridium mastoideum A. Massal., Symmict. Lich. 82, 1855. Basionym.

#### Type.

[Italy,] in op. Tregnago – Viacara (VER!, syntype).

#### Description.

Prothallus not seen. Thallus pale brownish-grey, continuous to rimose. Perithecia 0.12–0.21 mm, 3/4–immersed in thallus. Involucrellum apical, ca. 40–50 mm thick, appressed to the exciple. Exciple 0.27–0.33 mm in diam., wall black. Periphysoids ca. 40–45 × 2 mm. Ascospores 0-septate, 28–31 × 12–15 mm.

#### Notes.

The syntype specimen studied is probably conspecific with *V. viridula*.

### Verrucaria mimicrans

Taxon classificationFungiVerrucarialesVerrucariaceae

Servít, Stud. Bot. Čech. 11: 116, 1950

026178C5-CD4C-5CC4-B680-B0E7F102317C

#### Type.

?, protologue: “Jugoslavia, Pulac pr. Rijeka (Fiume), 250 m, dolom., Schuler (P)”.

#### Notes.

The type material was not located. *Verrucaria mimicrans* was reported from Finland by Pykälä and Breuss (2008), but the specimen belongs to *V. subtilis*. In the original description, the spore size of *V. mimicrans* is 25–31 × 12–15 mm (Servít 1950) which exceeds the values of *V. subtilis*. Thus, *V. mimicrans* may be distinct from *V. subtilis*, but not present in Finland.

### Verrucaria montenegrina

Taxon classificationFungiVerrucarialesVerrucariaceae

Servít, Stud. Bot. Čech. 9: 94, 1948

77E8F07F-6852-506D-8C29-9F20217A9291

#### Type.

[Montenegro,] Lovcen, Veterni mlin, 1400 m, 1929, M. Servít (PRM-859152!, holotype).

#### Description.

Prothallus not seen. Thallus grey with frequent tiny brown flecks, endolithic. Perithecia 0.18–0.26 mm, 3/4(–1)-immersed, leaving deep pits in the rock; ca. 60–80 perithecia/cm^2^. Involucrellum reaching the exciple base or enveloping the exciple, in the latter case diffusely pigmented under the exciple, ca. 70–110 mm thick, appressed to the exciple. Exciple ca. 0.20–0.22 mm in diam., wall dark. Periphysoids ca. 20–25 × 2.5–3 mm. Ascospores 0-septate (only few seen), 20–25 × 11–14 mm.

#### Notes.

The species differs from the species of the *V. subtilis* complex by thicker involucrellum and shorter spores. *Verrucaria samosensis* Servít has thinner involucrellum and shorter spores.

### Verrucaria moravica

Taxon classificationFungiVerrucarialesVerrucariaceae

Servít, Stud. Bot. Čech. 9: 95, 1948

BA9128FC-33D2-51E7-9905-519E7E25A329

#### Type.

[Czech Republic,] Moravia, Kopřivnice, Piskovnice, 490 m alt., 1922, Suza (PRM-760594!, syntype).

#### Description.

Prothallus not seen. Thallus whitish-grey with abundant medium greenish-brown punctae, endolithic, a dark line between contiguous conspecific thalli. Perithecia 0.23–0.35 mm, 3/4–1-immersed, leaving deep pits in the rock, surrounded by a thalline collar; ca. 40–60 perithecia/cm^2^. Involucrellum apical. Exciple ca. 0.26 mm in diam., wall brown. Ascospores 0-septate, 20–25 × 9–12 mm.

#### Notes.

Perithecia are mostly over-mature. One perithecium was sectioned. *V. moravica* may be rather similar to *V. subtilis*, but differs in the presence of dark lines between contiguous conspecific thalli. In the original description, the spore length was reported to be more variable: 18–28 × 9–12 (Servít 1948). In *V. transfugiens* Zschacke, the involucrellum is absent.

### Verrucaria muelleriana

Taxon classificationFungiVerrucarialesVerrucariaceae

Servít, Věstn. Krá. České Společ. Nauk 10: 14 (1948) [1947]

DA474CE5-8E7F-5AC9-86E9-79C81D538036

#### Type.

[France] Salève, J. Müller (M-0193432!, holotype).

#### Description.

Prothallus not seen. Thallus pale brown with a violet tinge, continuous, hemi-endolithic, contiguous conspecific thalli separated by dark lines. Perithecia 0.38–0.46 mm, 3/4-immersed, leaving deep pits in the rock; ca. 30–50 perithecia/cm^2^. Involucrellum apical, ca. 50–60 mm thick, appressed to the exciple. Exciple ca. 0.34–0.35 mm in diam., wall black, ca. 25 mm thick. Periphysoids ca. 50–80 × 1–1.5 mm. Ascospores 0-septate, 32–41 × 12–15 mm.

#### Notes.

The species is morphologically close to *V. depressula* or may even be conspecific.

### Verrucaria nylanderiana

Taxon classificationFungiVerrucarialesVerrucariaceae

Servít, Stud. Bot. Čech. 9: 96, 1948

82220A5D-3F6E-5AB2-93A1-48FA33618B28

#### Type.

[France] Gallia, Sevres (M-0193237!, holotype).

#### Description.

Prothallus not seen. Thallus greenish-grey with green flecks, continuous, ca. 0.1–0.3 mm thick. Perithecia 0.12–0.32 mm, immersed, leaving deep pits in the rock; ca. 80–100 perithecia/cm^2^. Involucrellum absent. Exciple ca. 0.27–0.41 mm in diam., higher than broad, often pear-shaped, wall dark brown. Periphysoids ca. 40–60 × 2–2.5 mm, branched-anastomosing. Asci 85–106 × 25–29 mm, 8-spored. Ascospores 0-septate, 18–23 × 12–14 mm.

#### Notes.

The species differs from *V. viridula* by shorter spores and absence of an involucrellum.

### Verrucaria oligocarpa

Taxon classificationFungiVerrucarialesVerrucariaceae

Servít, Stud. Bot. Čech. 9: 97, 1948

94BD649C-3B5F-5CB5-AFB0-C84570461469

#### Type.

[Germany] Eichstätt, ober dem Tiefenthale, 2. 1887, Boll (M-0204594!, holotype).

#### Description.

Prothallus not seen. Thallus grey, endolithic. Perithecia 0.08–0.21 mm, immersed, leaving deep pits in the rock; ca. 60–100 perithecia/cm^2^. Involucrellum absent. Exciple ca. 0.19–0.24 mm in diam., wall medium brown to dark brown, apex often thickened. Periphysoids ca. 15–30 × 2–2.5 mm. Asci ca. 61–69 × 20–21 mm, 8-spored. Ascospores 0-septate, 18–23 × 8–11 mm.

#### Notes.

The species may differ from *V. caesiopsila* by narrower spores and shorter periphysoids. *Verrucaria koerberi* has an apical involucrellum and narrower spores.

### Verrucaria pallidocarpa

Taxon classificationFungiVerrucarialesVerrucariaceae

Servít, Stud. Bot. Čech. 9: 98, 1948

DA3FA938-0E01-54FE-847A-DFD7612D15D7

#### Type.

Jugoslavia, Lovčen, Sanatorium, 1240 m, 1929, M. Servít (PRM-858454!, holotype?).

#### Description.

Prothallus not seen. Thallus grey with brown punctae, endolithic, contiguous conspecific thalli separated by dark lines. Perithecia 0.15–0.2 mm, 3/4(–1)-immersed, leaving deep pits in the rock; ca. 80–240 perithecia/cm^2^. Involucrellum absent. Exciple ca. 0.21–0.24 mm in diam., wall pale brown to medium brown, apex thickened to ca. 40–50 mm thick. Ascospores 0-septate 16–24 × 10–13(–14) mm.

#### Notes.

The species is rather similar to *V. transfugiens*, but has a paler exciple wall and slightly larger spores.

### Verrucaria paradolomitica

Taxon classificationFungiVerrucarialesVerrucariaceae

Servít, Stud. Bot. Čech. 9: 99, 1948

71D3E76E-375B-5F44-B271-3E5126B94A5F

#### Type.

[Austria,] Dolomit … Grosser Rettenstein bei Kizbühel im Tirol, 1869, Arnold (PRM-858456!, isotype).

#### Description.

Prothallus not seen. Thallus pale brown, epilithic, thin, continuous. Perithecia 0.15–0.23 mm, (3/4–)1-immersed, leaving deep pits in the rock. Involucrellum absent or apical, ca. 70–90 mm thick. Exciple ca. 0.22–0.25 mm in diam., wall blackish-brown, the apex is strongly thickened when the involucrellum is absent. Ascospores 0-septate, 27–37 × 12–15 mm.

#### Notes.

The species may fall within the variation of *V. foveolata*, although it has an epilithic pale brown thallus.

### Verrucaria periphysata

Taxon classificationFungiVerrucarialesVerrucariaceae

Zahlbr., Österr. Bot. Zeitschrift 68: 67, 1919

65EFF958-D25C-5380-B62D-E4D86CAD94A9

#### Type.

[Croatia] Dalmatien: Schlossruine Vrlika a.d. … Granuga, an Kalk… c. 550 m, 5.7.1911, J. Baumgartner 4250 (W-4250!).

#### Description.

Prothallus not seen. Thallus endolithic, grey. Perithecia 0.15–0.34 mm, immersed, leaving deep pits in the rock; ca. 100–130 perithecia/cm^2^. Involucrellum absent. Exciple ca. 0.35–0.5 mm in diam., longer than wide, often pear-shaped, apex thickened, wall black. Periphysoids ca. 50–80 × 2 mm. Ascospores 0-septate, 26–35 × 12–14 mm.

#### Notes.

Material similar to *V. periphysata* has not been observed in Finland. The exciple of the species is larger than in *V. foveolata* (0.2–0.4 mm in diam.). The periphysoids may also be longer.

### Verrucaria praecellens

Taxon classificationFungiVerrucarialesVerrucariaceae

(Arnold) Servít, Stud. Bot. Čech. 9: 100, 1948

76D9B32A-82B8-59A4-8BE4-60738257E451

Amphoridium praecellens Arnold, Verh. Zool. Bot. Ges. 19: 651, 1869. Basionym.

#### Type.

[Italy] 87. Dolomitfelsen in der Schlernklamm ober … in Süd Tirol, 7.1867, Arnold (H-NYL 3208!, H-NYL 3209!, syntypes).

#### Description.

Prothallus absent. Thallus endolithic, grey with a violet tinge, a dark line between contiguous conspecific thalli present, 0.15–0.22 mm wide. Perithecia 0.21–0.44 mm, immersed in rock, leaving deep pits in the rock. Ostiolar depression large. Involucrellum absent or possibly in some perithecia, small apical involucrellum. Exciple ca. 0.4 mm in diam., apex thickened to ca. 60–80 mm, wall black. Periphysoids ca. 50–60 × 2 mm. Ascospores 0-septate, ca. 26–34 × 16–20 mm, perispore ca. 1–1.5 mm thick.

#### Notes.

The perithecia of the syntypes in H-NYL are mainly over-mature. The spore size annotated by Nylander to the specimen is larger (40–48 × 23–26 mm) than the few spores measured by us. Servít (1948) reported high variation in the spore size: 20–45 × 18–26 mm. *Verrucaria praecellens* seems to be characterised by broad spores and a persistent perispore. *Verrucaria cryptica* has narrower spores. *V. praecellens* has been synonymised with *V. hochstetteri* (Krzewicka 2012), but it may differ by persistent perispores and larger spores.

### Verrucaria pustulifera

Taxon classificationFungiVerrucarialesVerrucariaceae

Servít, Stud. Bot. Čech. 11(3): 120, 1950

2C5B4C7B-4933-519E-87A9-EBE54C627AE0

#### Type.

Slovakia, in valle fl. Hnilec, pr. R. Ztratená, 800 m alt., calc., 1933, Suza (PRM 858074!, syntype).

#### Description.

Prothallus not seen. Thallus grey, endolithic to semi-endolithic. Perithecia 0.25–0.33 mm, 3/4-immersed, leaving deep pits in the rock, usually surrounded by a thalline collar or is covered by a thin thalline layer except for the apex. Ostiole pale, plane, ca. 20–50 mm wide. Involucrellum covering half of the exciple, ca. 50–70 mm thick, diverging from the exciple. Exciple ca. 0.3–0.33 mm in diam., wall pale brown. Periphysoids ca. 25–30 × 2–2.5 mm. Ascospores 0-septate, 27–38 × 12–15 mm.

#### Notes.

*Verrucaria pustulifera* differs from *V. subjunctiva* in a pale brown exciple and shorter periphysoids. The involucrellum is also smaller than usually in *V. subjunctiva*. *Verrucaria kuusamoensis* has smaller spores.

### Verrucaria reculetensis

Taxon classificationFungiVerrucarialesVerrucariaceae

Servít, Stud. Bot. Čech. 11(3): 103, 1950

8CD6956A-C846-54C8-A982-46644C40024E

#### Type.

[France] Reculet, Jan. 1855, J. Müller (M-0220250!, holotype).

#### Description.

Prothallus not seen. Thallus pale brown, epilithic, thin, continuous. Perithecia 0.38–0.55 mm, 1/2–3/4-immersed, leaving deep pits in the rock; ca. 30–40 perithecia/cm^2^. Ostiole tiny, inconspicuous, dark, plane, often surrounded by a projecting neck up to ca. 150 mm wide. Involucrellum absent. Exciple ca. 0.38–0.45 mm in diam., wall dark, ca. 30–40 mm thick, apex thickened to 70–100 mm thick. Periphysoids ca. 50–60 × 1.5–2 mm, branched-anastomosing. Ascospores 0-septate, 25–30 × 13–16 mm.

#### Notes.

The species is rather similar to *V. foveolata*, but may differ by slightly larger perithecia and an epilithic pale brown thallus.

### Verrucaria samosensis

Taxon classificationFungiVerrucarialesVerrucariaceae

Servít, Stud. Bot. Čech. 9: 105, 1948

36E86F67-D65C-5A5A-AD7F-E054E6CBF2B8

#### Type.

[Greece,] Samos, Vathy, Rechinger (PRM-858434!, holotype).

#### Description.

Prothallus not seen. Thallus whitish-grey, endolithic to thinly epilithic, occasionally irregularly rimose around perithecia. Perithecia 0.22–0.28 mm, (1/2–)3/4-immersed, leaving shallow to deep pits in the rock; ca. 70–80 perithecia/cm^2^. Involucrellum enveloping the exciple, 40–50 mm thick. Exciple 0.19–0.28 mm in diam., wall black. Periphysoids ca. 50–60 × 2–2.5 mm. Ascospores 0-septate, ca. 21–25 × 11–13 mm.

#### Notes.

According to the protologue, the spores may be larger: 20–29 × 9–15 mm (Servít 1948). The species differs from *V. bifurcata* by longer periphysoids and possibly by slightly shorter, but broader spores.

### Verrucaria saprophila

Taxon classificationFungiVerrucarialesVerrucariaceae

(A. Massal.) Trevis., Conspect. Verruc.: 8, 1860

1A9D83B1-E144-56FF-9682-00B5139B46D8

Amphoridium saprophilum A. Massal., Symmicta Lich. 79, 1855. Basionym.

#### Type.

[Italy,] avi in op. Avesa ([Monte] Ongarine) ad saxa putrida eocenica (VER!, syntype).

#### Description.

Prothallus not seen. Thallus whitish-grey, endolithic to semi-endolithic, a black line between thalli present. Perithecia 0.16–0.23 mm, immersed, leaving deep pits in the rock. Involucrellum absent. Exciple ca. 0.26 mm in diam., wall brown. Ascospores 0-septate, 24–33 × 12–18 mm.

#### Notes.

Krzewicka (2012) treated *V. saprophila* as a synonym of *V. hochstetteri*. However, *V. hochstetteri* has a larger exciple and broader spores. The species differs from *V. foveolata* with the presence of a dark line. *Verrucaria dolomitica* has larger perithecia and an apical involucrellum.

### Verrucaria sbarbaronis

Taxon classificationFungiVerrucarialesVerrucariaceae

de Lesd., Bull. Soc. Bot. Fr. 94: 199, 1948

FB196BFE-72BD-5330-ACB8-03208F599B73

#### Type.

Not seen. Protologue: “Italia, in Valle Bisagno prope Genuam, loco Prato, supra rupem calcaream colore fuscorufo tinctam. leg. Sbarbaro, 1946”.

#### Notes.

The type material of *V. sbarbaronis* has not been located. Breuss (2008a) treated *V. sbarbaronis* as a species rather similar to *V. lacerata* (which is here considered conspecific with *V. subjunctiva*), but with clearly smaller spores (20–26 × 11–15 mm). Such specimens have not been found from Finland.

### Verrucaria serlosensis

Taxon classificationFungiVerrucarialesVerrucariaceae

Servít, Stud. Bot. Čech. 9: 106, 1948

E7E2A352-6D9F-5E93-B969-F053A08C8DEA

#### Type.

[Austria], Kalksteine des Serlosgipfels 8200’ Matrei-Tirol, 7. 1869, Arnold (M-0193173!, holotype).

#### Description.

Prothallus not seen. Thallus grey to pale brown, endolithic, somewhat inconspicuous. Perithecia 0.12–0.22 mm, immersed, leaving deep pits in the rock; ca. 60–100 perithecia/cm^2^. Involucrellum absent. Exciple ca. 0.2 mm in diam., wall pale to pale brown, apex dark, thickened. Ascospores 0-septate, (23.2–)23.9–24.7–25.4(–25.5) × (12.7–)12.8–13.7–14.6(–15.1) mm (n = 15).

#### Notes.

The specimen is rather poor. The species differs from *V. foveolata* by a pale exciple wall, shorter spores and perhaps by a smaller exciple. *Verrucaria caesiopsila* has smaller spores and a dark exciple wall.

### Verrucaria slovaca

Taxon classificationFungiVerrucarialesVerrucariaceae

Servít, Stud. Bot. Čech. 11: 125, 1950

95169723-A183-5F80-8EB5-324A6CFBBABA

#### Type.

Slovakia, Liptovskě hole, Zuberec, Osobita, 1650–1680 m, 1935, Suza (PRM-765231!, syntype).

#### Description.

Prothallus not seen. Thallus white or grey, endolithic. Perithecia 0.25–0.35 mm, 1/2–3/4(–1)-immersed, leaving shallow to deep pits in the rock. Involucrellum reaching the exciple base, ca. 70–90 mm thick, appressed to the exciple or slightly diverging from the exciple. Exciple ca. 0.15–0.25 mm in diam., wall pale. Periphysoids ca. 25 × 2–2.5 mm. Ascospores 0-septate, few spores 1-septate, ca. 20–27 × 9–10 mm, not well developed.

#### Notes.

*Verrucaria slovaca* may possibly belong to the *V. subtilis* complex, but similar specimens have not been found in Finland. The spore size given by Servít (1950) is 24–30 × 9–11 mm. Breuss (2016) apparently found better-developed spores than in this study: (22–)24–27(–28) × (7.5–)9–11–(12) mm.

### Verrucaria strasseri

Taxon classificationFungiVerrucarialesVerrucariaceae

Servít, Stud. Bot. Čech. 9: 107, 1948

21F4BC43-2CF6-5956-8CC1-7A50403C5A64

#### Type.

[Italy], Auf Kalkconglommerat in Villa Lagarina bei Roveredo in Südtirol, 1.5.1883, P. Strasser (M-02039301!, holotype).

#### Description.

Prothallus not seen. Thallus whitish-grey, endolithic. Perithecia 0.15–0.38 mm, (3/4)–1-immersed, leaving deep pits in the rock. Involucrellum apical, ca. 50–90 mm thick, appressed to the exciple. Exciple ca. 0.22–0.26 mm in diam., wall pale brown to dark brown. Periphysoids ca. 30–50 × 1.5–2.5 mm, branching. Asci ca. 88–95 × 26–28 mm, 8-spored. Ascospores 0-septate, (23.6–)24.5–26.6–28.8(–30.2) × (10.1–)10.4–11.4–12.4(–13.7) mm (n = 17).

#### Notes.

The species may differ from the *V. devergens* and *V. subtilis* complexes by mostly fully immersed perithecia and from the *V. subtilis* complex by slightly larger perithecia and a thicker involucrellum.

### Verrucaria transfugiens

Taxon classificationFungiVerrucarialesVerrucariaceae

Zschacke, Rabenh. Krypt.-Fl. 9, 1(1): 85, 1933

3D11F9BD-AD99-53C5-8A72-9498F6A7E236

#### Type.

Deutschland. Thüringen, Jonastal bei Arnstadt, alt. 350–400 m, co-ord. 10°55'E, 50°49'N. An Muschelkalkplättchen, 7.7.1907, G. Lettau (B-600025730!); Deutschland.Sachsen-Anhalt: Vorland des Nord-Ost-Harzes, Steinbruch am Hackel. co-ord. 11°19'E, 51°53'N, 1910, H. Zschacke 4664 (B-600194785!); Deutschland.Sachsen-Anhalt: Harz-Vorland, Ostseite des Hackels. co-ord. 11°19'E, 51°53'N, 1.2.1906, H. Zschacke 4664 (B-600194786!); Deutschland. Thüringen: Dosdorfer Haart, unweit Arnstadt, alt. 450 m, an Muschel Kalk-Felsbänken, accomp. *Tichothecium erraticum*, *Caloplaca lactea* 11.9.1907, G. Lettau 614 (B-600194783!); Deutschland. Thüringen: Dosdorfer Haart, unweit Arnstadt, alt. 450 m, an Muschel Kalk-Felsbänken, 1907?, G. Lettau (B-600194781!). Syntypes.

For the description of the species, see Pykälä (2016). *V. transfugiens* has been reported from Finland by Pykälä and Breuss (2008), but the specimens belong to *V. subtilis* (Stenroos et al. 2016).

### Verrucaria veronensis

Taxon classificationFungiVerrucarialesVerrucariaceae

A. Massal., Ric. Auton. Lich. Crost. 173, 1852

827973DD-FDF7-5A5B-B006-8C71D57B50C6

#### Type.

[Italy,] S. Leonardo, L. Tonini (VER!, syntype); ad saxa eocenica circa urbem Veronam (S. Leonardo), leg. Tonini, Massalongo Lichenes Ital. Exsiccatae 8 (VER!, syntype); Massalongo, Lich. Ital. exs. 8 (UPS!, syntype).

#### Description.

Prothallus absent. Thallus greenish-grey or grey with some brown pigmentation, epilithic, rimose, ca. 0.2–0.3(–0.4) mm thick. Perithecia 0.12–0.32 mm, 3/4–1-immersed in thallus. Involucrellum apical, ca. 60–70 mm thick. Exciple ca. (0.2–)0.3–0.5 mm in diam., often longer than broad, wall dark. Ascospores 0-septate, 27–35 × 11–15 mm.

#### Notes.

The type material of the species is morphologically similar to *V. viridula* and, based on the morphological similarity, the species is likely to be conspecific with *V. viridula*.

## Supplementary Material

XML Treatment for Verrucaria bifurcata

XML Treatment for Verrucaria cavernarum

XML Treatment for Verrucaria devergens

XML Treatment for Verrucaria difficilis

XML Treatment for Verrucaria foveolata

XML Treatment for Verrucaria fuscozonata

XML Treatment for Verrucaria karelica

XML Treatment for Verrucaria kuusamoensis

XML Treatment for Verrucaria subdevergens

XML Treatment for Verrucaria subjunctiva

XML Treatment for Verrucaria subtilis

XML Treatment for Verrucaria vacillans

XML Treatment for Verrucaria adelminienii

XML Treatment for Verrucaria aljazevi

XML Treatment for Verrucaria alpigena

XML Treatment for Verrucaria bavarica

XML Treatment for Verrucaria caesiopsila

XML Treatment for Verrucaria carnea

XML Treatment for Verrucaria cinereorufa

XML Treatment for Verrucaria clauzadei

XML Treatment for Verrucaria cryptica

XML Treatment for Verrucaria depressula

XML Treatment for Verrucaria dermatoidea

XML Treatment for Verrucaria dolomitica

XML Treatment for Verrucaria epipolaea

XML Treatment for Verrucaria euboensis

XML Treatment for Verrucaria grossa

XML Treatment for Verrucaria hercegensis

XML Treatment for Verrucaria hochstetteri

XML Treatment for Verrucaria integra

XML Treatment for Verrucaria integrella

XML Treatment for Verrucaria koerberi

XML Treatment for Verrucaria mastoidea

XML Treatment for Verrucaria mimicrans

XML Treatment for Verrucaria montenegrina

XML Treatment for Verrucaria moravica

XML Treatment for Verrucaria muelleriana

XML Treatment for Verrucaria nylanderiana

XML Treatment for Verrucaria oligocarpa

XML Treatment for Verrucaria pallidocarpa

XML Treatment for Verrucaria paradolomitica

XML Treatment for Verrucaria periphysata

XML Treatment for Verrucaria praecellens

XML Treatment for Verrucaria pustulifera

XML Treatment for Verrucaria reculetensis

XML Treatment for Verrucaria samosensis

XML Treatment for Verrucaria saprophila

XML Treatment for Verrucaria sbarbaronis

XML Treatment for Verrucaria serlosensis

XML Treatment for Verrucaria slovaca

XML Treatment for Verrucaria strasseri

XML Treatment for Verrucaria transfugiens

XML Treatment for Verrucaria veronensis
