# Corrigendum: Pykälä J, Kantelinen A, Myllys L (2020) Taxonomy of *Verrucaria* species characterised by large spores, perithecia leaving pits in the rock and a pale thin thallus in Finland. MycoKeys 72: 43–92. https://doi.org/10.3897/mycokeys.72.56223

**DOI:** 10.3897/mycokeys.80.67870

**Published:** 2021-06-03

**Authors:** Juha Pykälä, Annina Kantelinen, Leena Myllys

**Affiliations:** 1 Biodiversity Centre, Finnish Environment Institute, Latokartanonkaari 11, 00790, Helsinki, Finland Biodiversity Centre, Finnish Environment Institute Helsinki Finland; 2 Botanical Museum, Finnish Museum of Natural History, P.O. Box 7, FI-00014, University of Helsinki, Finland University of Helsinki Helsinki Finland

## Abstract

Corrigendum

Almost all micrometres (μm) have been erroneously changed into millimetres (mm). All measurements of algal cells, ostiole, involucrellum thickness, exciple wall thickness, periphysoids, asci, ascospores and perispores should be in micrometres (μm). Furthermore, thallus thickness should be in micrometres in *Verrucaria bifurcata*, *V. cavernarum*, *V. difficilis* and *V. vacillans*.

In the lectotypification of *Verrucaria subjunctiva* registration number MBT392473, MBT is missing from the MycoBank number. This note validates the lectotypification.

## Verrucaria subjunctiva

Taxon classificationFungiVerrucarialesVerrucariaceae

Nyl., Flora 67: 218, 1884

74AA9698-C3B0-55FD-A505-D2DD371AC460

392473

### Type.

[RUSSIA], Sibiria Septentrionalis: Sinus Konyam ad fretum Bering, 64°50 lat. bor., 173° long. occid. (Greenw.) 28–30.7.1879 E. Almquist (S-L46!, lectotype, designated here); Fretum Behring, Konyam Bay, E. Almquist (H-NYL 3512!, isolectotype).

## Supplementary Material

XML Treatment for Verrucaria subjunctiva

